# Mitochondrial uncoupler BAM15 reverses diet-induced obesity and insulin resistance in mice

**DOI:** 10.1038/s41467-020-16298-2

**Published:** 2020-05-14

**Authors:** Stephanie J. Alexopoulos, Sing-Young Chen, Amanda E. Brandon, Joseph M. Salamoun, Frances L. Byrne, Christopher J. Garcia, Martina Beretta, Ellen M. Olzomer, Divya P. Shah, Ashleigh M. Philp, Stefan R. Hargett, Robert T. Lawrence, Brendan Lee, James Sligar, Pascal Carrive, Simon P. Tucker, Andrew Philp, Carolin Lackner, Nigel Turner, Gregory J. Cooney, Webster L. Santos, Kyle L. Hoehn

**Affiliations:** 1 School of Biotechnology and Biomolecular Sciences, University of New South Wales, Sydney, NSW 2052 Australia; 2 Sydney Medical School, Charles Perkins Centre, University of Sydney, Sydney, NSW 2006 Australia; 3 Department of Chemistry and Virginia Tech Centre for Drug Discovery, Virginia Tech, Blacksburg, VA 24061 USA; 4 Garvan Institute of Medical Research, Darlinghurst, NSW Australia; 5 Department of Pharmacology, University of Virginia, Charlottesville, VA 22908 USA; 6 Biological Resources Imaging Laboratory, University of New South Wales, Sydney, NSW 2052 Australia; 7 Department of Anatomy, School of Medical Sciences, University of New South Wales, Sydney, NSW 2052 Australia; 8 Continuum Biosciences Pty Ltd., Sydney, NSW 2035 Australia; 9 Institute of Pathology, Medical University of Graz, Graz, Austria; 10 Department of Pharmacology, School of Medical Science, University of New South Wales, Sydney, NSW 2052 Australia

**Keywords:** Obesity, Metabolic syndrome, Obesity

## Abstract

Obesity is a health problem affecting more than 40% of US adults and 13% of the global population. Anti-obesity treatments including diet, exercise, surgery and pharmacotherapies have so far failed to reverse obesity incidence. Herein, we target obesity with a pharmacotherapeutic approach that decreases caloric efficiency by mitochondrial uncoupling. We show that a recently identified mitochondrial uncoupler BAM15 is orally bioavailable, increases nutrient oxidation, and decreases body fat mass without altering food intake, lean body mass, body temperature, or biochemical and haematological markers of toxicity. BAM15 decreases hepatic fat, decreases inflammatory lipids, and has strong antioxidant effects. Hyperinsulinemic-euglycemic clamp studies show that BAM15 improves insulin sensitivity in multiple tissue types. Collectively, these data demonstrate that pharmacologic mitochondrial uncoupling with BAM15 has powerful anti-obesity and insulin sensitizing effects without compromising lean mass or affecting food intake.

## Abstract (web summary)

Obesity is a global pandemic with limited treatment options. Here, the authors show evidence in mice that the mitochondrial uncoupler BAM15 effectively induces fat loss without affecting food intake or compromising lean body mass.

## Introduction

Obesity is associated with shortened lifespan and contributes to complications including infertility, mental health problems and metabolic diseases including cardiovascular diseases, fatty liver disease, and at least 13 types of cancer^1–6^. Identification of drugs that safely reverse obesity could increase healthspan and improve quality of life on a global scale.

Exercise and calorie restriction are effective anti-obesity interventions, but few patients adhere to the life-long diet and exercise programs required^7–9^. For example, one population-based cohort study of ~300,000 participants over a 9-year period found that the probability of an obese person, with a body mass index (BMI > 30), attaining a normal weight (BMI < 25) was <1%, while for morbidly obese individuals (BMI > 40) this incidence decreased to ~0.1%^10^. Bariatric surgery is the most effective anti-obesity intervention^11,12^ but it is not a viable global solution due to the cost and risks involved^13,14^. There is clear need for pharmacological weight-loss assistance that can be used alone or as an adjunct to lifestyle modification.

There are several FDA-approved anti-obesity drugs that primarily target food intake or nutrient absorption, but most have unwanted on- and off-target adverse effects that prevent their mainstream adoption; reviewed in Manning et al.^15^ and Dietrich and Horvath^16^. An alternative approach to decreasing food intake or absorption is to decrease the metabolic efficiency whereby food is converted into useful energy^17–19^. Small molecule mitochondrial protonophore uncouplers decrease mitochondrial coupling efficiency resulting in increased nutrient oxidation to produce a given amount of ATP^17^. The mitochondrial uncoupler 2,4-dinitrophenol (DNP) shows proof-of-principle that mitochondrial protonophores have weight-loss effects in humans as ~90% of patients taking 300 mg DNP per day (3 mg/kg for a 100 kg person) lost 2–3 pounds per week without changes in food intake^20,21^. However, DNP has a narrow window between effective and toxic doses^22,23^ and it was banned for human use by the FDA in 1938. To overcome the narrow therapeutic window of DNP, researchers have developed less-toxic derivatives that are targeted to mitochondria, targeted to the liver, or formulated DNP for controlled-release. Targeting DNP to mitochondria by attaching lipophilic cations was ineffective because DNP could not recycle across the mitochondrial inner membrane^24^; however, liver-targeted DNP prodrugs and controlled-release formulations resulted in considerably improved safety and beneficial effects on insulin sensitivity and fatty liver disease^25,26^. Unfortunately, these approaches to control DNP toxicity resulted in the loss of anti-obesity effects. In fact, DNP is not well tolerated in mice and doses up to 89 mg/kg/d administered in drinking water did not result in weight loss in mice housed at room temperature^27^.

More than a dozen classes of molecules have mitochondrial protonophore activity, but molecules with activity specific to mitochondria are rare and only a few have anti-obesity efficacy with a suitable therapeutic window, reviewed in refs.^28,29^. In this study, we investigate the anti-obesity potential of BAM15 (N5,N6-bis(2-Fluorophenyl)[1,2,5]oxadiazolo[3,4-b]pyrazine-5,6-diamine), a recently-identified mitochondrial protonophore uncoupler that is structurally unrelated to DNP. We have previously validated BAM15 as a lipophilic weak acid mitochondrial protonophore, demonstrated that it can drive maximal mitochondrial respiration in vitro over a broad dosing range without causing respiratory collapse, and showed that intraperitoneal injection of BAM15 protected mice from acute kidney injury caused by ischemia–reperfusion^30,31^.

Herein we show that BAM15 is orally bioavailable, dose-dependently increases nutrient oxidation, and decreases body fat mass without altering food intake or decreasing lean body mass in mice fed an obesigenic western diet (WD). We show that BAM15 decreases insulin resistance in multiple tissue types and has beneficial effects on liver fat, oxidative stress, and inflammation. These phenotypes occur with no observed adverse effects detected in body temperature or key clinical biochemistry and haematology parameters.

## Results

### BAM15 properties and biological activity

**Fig. 1:**
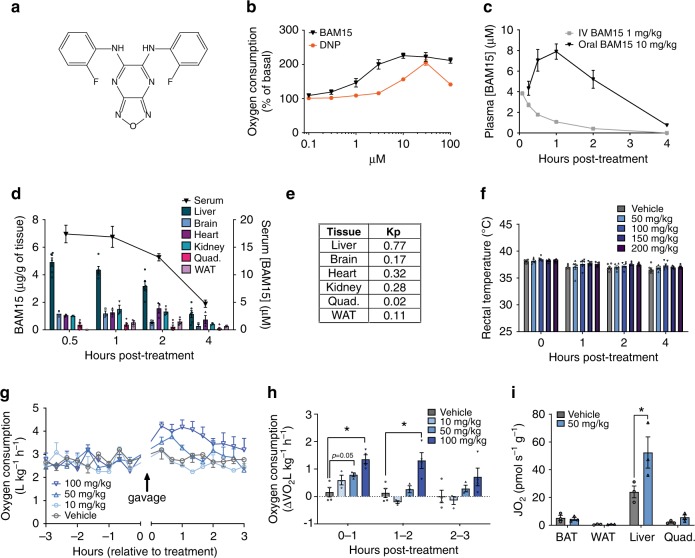
Properties and biological activity of BAM15. **a** Chemical structure of BAM15. **b** Oxygen consumption rate (OCR) of normal mouse liver cells (NMuLi) treated with BAM15 (black triangles) or DNP (orange squares), measured by Seahorse XF assay, *n* = 3 biologically independent experiments. **c** Pharmacokinetics of BAM15 administered by i.v. injection at 1 mg/kg (*n* = 4 animals) and oral gavage at 10 mg/kg (*n* = 3 animals). **d** Tissue distribution and (**e**) plasma partition coefficient (*K*p) of BAM15 administered by oral gavage at 50 mg/kg, *n* = 6 animals for liver, quad and serum examined over two independent experiments, *n* = 3 animals used for brain, heart, kidney, WAT measurements, (*n* = 2 for brain 0.5 h timepoint only). **f** Body temperature measured over a 4 h period following oral gavage with vehicle or BAM15 at indicated doses. Significance tested with two-tailed Student’s *t* test comparing treatment to vehicle control at each timepoint, *n* = 6 animals. **g**, **h** Whole-body oxygen consumption rate measured in mice administered vehicle or BAM15 by oral gavage at indicated doses. Changes in oxygen consumption rate were quantified as delta VO_2_ from baseline for the periods 0–1, 1–2 and 2–3 h after oral gavage. *n* = 3 animals for 10 mg/kg, 50 mg/kg and *n* = 4 animals for vehicle and 100 mg/kg. Asterisk (*) indicates *p* < 0.004 by one-way ANOVA. **i** Tissue specific oxygen consumption measured by OROBOROS assay, 1 h post-oral gavage of 50 mg/kg BAM15 or vehicle control, *n* = 3 animals, asterisk (*) indicates *p* = 0.001 by two-way ANOVA. All data presented as mean values ± SEM. (**f**–**i**) Colour scale: grey—vehicle, pale blue—10 mg/kg, light blue—50 mg/kg, electric blue—100 mg/kg, navy blue—150 mg/kg, dark purple—200 mg/kg.

We first compared BAM15 (Fig. 1a) to the prototype mitochondrial uncoupler DNP using a Seahorse XF Analyser to assess drug-induced oxygen consumption rate. Normal murine liver (NMuLi) cells were treated with BAM15 or DNP at doses ranging from 0.1 to 100 μM (Fig. 1b). This experiment showed that BAM15 was ~7-fold more potent than DNP for stimulating oxygen consumption rate (OCR) (average EC_50_ values of 1.4 μM for BAM15 vs. 10.1 μM for DNP) and was capable of sustaining high levels of mitochondrial respiration across a broad concentration range from 3 to 100 µM (Fig. 1b). However, the dosing window where DNP stimulated maximal respiration was much smaller than for BAM15 and dose escalation of DNP above 30 µM resulted in respiratory inhibition (Fig. 1b). A similar dose response relationship was observed for each uncoupler on extracellular acidification rate (Supplementary Fig. 1).

We next assessed oral bioavailability of BAM15 in mice by comparing pharmacokinetics when delivered per oral (p.o.) or by intravenous (i.v.) tail vein injection. A dose-normalized area under the curve calculation showed that BAM15 was 67% orally bioavailable and p.o. delivery resulted in an average maximum plasma concentration (*C*_max_) of 8.2 µM with a 1.7 h half-life (*t*_1/2_) in C57BL/6J mice (Fig. 1c). BAM15 tissue distribution was determined by orally administering BAM15 (50 mg/kg) and euthanizing mice at times 0.5, 1, 2, and 4 h. BAM15 measurement in tissue extracts revealed primary distribution to the liver with gradual clearance from tissues over 4 h (Fig. 1d, e).

To determine the maximum range for acute dosing of BAM15 we assessed core body temperature using a rectal probe thermometer and measured key biochemical and haematological indicators of toxicity. We found that BAM15 dosing was limited by solubility in methylcellulose such that preparations for 100 mg/kg resulted in a fine suspension, while more concentrated solutions of 150 and 200 mg/kg resulted in a thickening paste. Nevertheless, doses up to 200 mg/kg had no effect on body temperature (Fig. 1f). Dosing was not escalated beyond 200 mg/kg because it became impractical to solubilize BAM15 for p.o. delivery and transient behavioural signs of lethargy were observed that were not possible to distinguish drug effects from delivery of paste to the stomach. DNP was highly soluble but resulted in a no observed adverse effect level of 25 mg/kg (Supplementary Fig. 2).

We next examined the pharmacodynamic effect of BAM15 by assessing oxygen consumption using an Oxymax CLAMS indirect calorimeter. Mice were orally administered BAM15 at doses of 10, 50, or 100 mg/kg, or vehicle control. The vehicle and 10 mg/kg BAM15 dose had no detectable impact on OCR while the 50 and 100 mg/kg doses of BAM15 increased oxygen utilisation in the first hour following gavage by 30% (*p* = 0.050, compared with vehicle control) and by 50% over baseline (*p* < 0.001, compared with vehicle control), respectively. In mice administered 100 mg/kg BAM15, increased OCR was sustained in the period 1–2 h post gavage by 48% over baseline (*p* = 0.004, compared with vehicle control), then gradually returned to baseline ~3 h post gavage (Fig. 1g, h), which is consistent with the pharmacokinetic profile (Fig. 1c).

To better understand which tissues were contributing to increased energy expenditure, we treated mice with 50 mg/kg BAM15 for 1 h and analysed OCR in homogenates from key metabolic tissues including liver, muscle, white fat and brown fat. Liver tissue from BAM15-treated mice had a 54% increase in respiration rate compared to the vehicle control, while muscle showed a non-significant trend for increased respiration and adipose tissue depot respiration was unchanged (Fig. 1i).

### BAM15 fed in Western diet increases energy expenditure

**Fig. 2:**
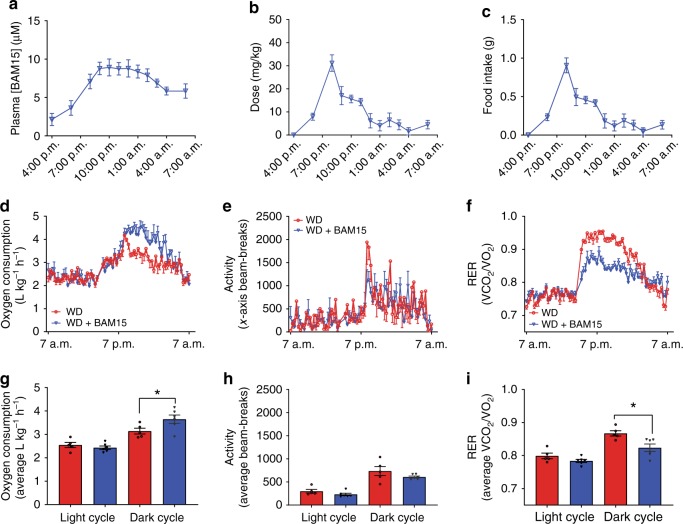
BAM15 increases energy expenditure. **a** Plasma drug exposure for mice fed WD containing 0.1% BAM15 (WD + BAM15) was measured overnight. **b** Dose of BAM15 calculated from (**c**) food intake, recorded overnight. **d** BAM15 feeding increased oxygen consumption rate by an average of 15% over the dark cycle, without altering (**e**) locomotor activity. **f** Mice fed WD + BAM15 had lower respiratory exchange ratios (RER) during the dark cycle. Average (**g**) oxygen consumption, (**h**) locomotor activity and (**i**) RER were used to calculate significant difference between treatment groups within each cycle, determined by two-tailed unpaired *t* test with Welch’s correction, asterisk (*) indicates *p* < 0.05, exact *p* values—(**g**) *p* = 0.0469, (**i**) *p* = 0.0128. *n* = 5 WD treated animals, *n* = 6 WD + BAM15-treated animals for all data. All data presented as mean values ± SEM. Colour scale: red—Western diet (WD), blue—Western diet + BAM15 (WD + BAM15). Source data are provided in Source data file.

BAM15 has low aqueous solubility and a half-life of 1.7 h; therefore, it was admixed in food for long-term treatment studies. A physiologically relevant high-fat (45% by kCal) high-sucrose (16% by kCal) WD was prepared with or without 0.1% w/w BAM15 (WD + BAM15). Mice were monitored for food intake and BAM15 exposure overnight. The 0.1% w/w BAM15 dose resulted in plasma concentrations between 5 and 10 µM for the entire dark period (7 p.m.–7 a.m.) (Fig. 2a), with the actual dose of BAM15 (Fig. 2b) calculated from food intake (Fig. 2c). We next assessed energy expenditure in mice fed WD + BAM15 compared with mice fed WD control (without BAM15) by indirect calorimetry. Relative to WD controls, mice fed 0.1% BAM15 in WD had an average 15% increase in oxygen consumption during the dark period (*p* < 0.001, Fig. 2d, g). The increased rate of energy expenditure correlated with drug exposure in the dark period and was not caused by changes in locomotor activity (Fig. 2e, h). The average respiratory exchange ratio (*V*CO_2_/*V*O_2_) was lower during the dark period in WD + BAM15 fed mice than controls (WD 0.86 ± 0.011 vs. WD + BAM15 0.82 ± 0.004, *p* = 0.010), indicating that BAM15 increased fat oxidation (Fig. 2f, i).

### BAM15 prevents diet-induced fat gain and glucose intolerance

**Fig. 3:**
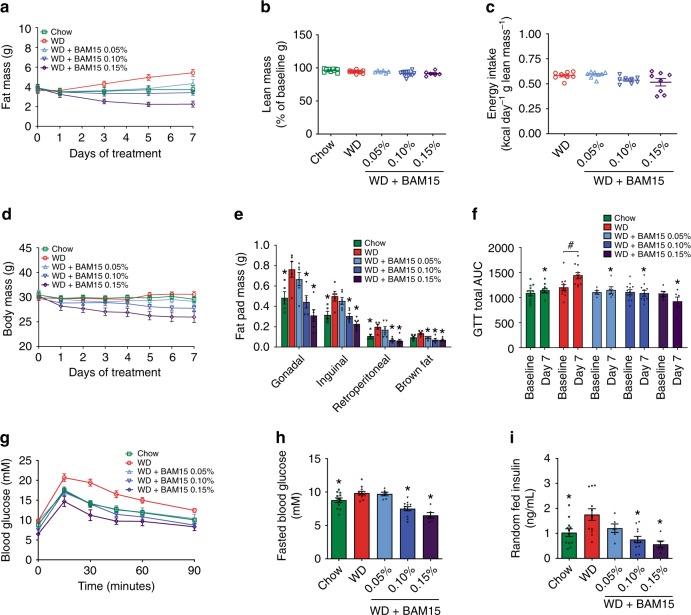
BAM15 prevents diet-induced fat gain and glucose intolerance. **a**–**d** Mice fed a chow diet or WD ± BAM15 at doses of 0.05, 0.10, and 0.15% were evaluated for changes in fat mass, lean mass, and food intake and body weight. *n* = 12 animals for Chow, WD, WD + BAM15 0.10%, data obtained from three independent studies, *n* = 6 animals for WD + BAM15 0.05% and WD + BAM15 0.15% data from one study. **e** Average fat pad masses dissected from mice after 8 days of treatment. *n* = 6 animals for all treatment groups, data from one study. Significance determined by one-way ANOVA, where asterisk (*) indicates *p* < 0.05 compared with WD. Exact *p* values as follows—Gonadal: Chow, *p* = 0.0321; WD + BAM15 0.10%, *p* = 0.0124; WD + BAM15 0.15%, *p* = 0.0003. Inguinal: Chow, *p* = 0.0133; WD + BAM15 0.10%, *p* = 0.008; WD + BAM15 0.15%, *p* = 0.0002. Retroperitoneal: Chow, *p* = 0.036; WD + BAM15 0.10%, *p* = 0.0019; WD + BAM15 0.15%, *p* = 0.0006. Brown fat: WD + BAM15 0.05%, *p* = 0.0199; WD + BAM15 0.10%, *p* = 0.0017; WD + BAM15 0.15%, *p* = 0.0008. **f**–**i** *n* = 12 animals for Chow, WD, WD + BAM15 0.10%, data obtained from three independent studies, *n* = 6 animals for WD + BAM15 0.05% and WD + BAM15 0.15% data from one study. **f** Intraperitoneal glucose tolerance test (i.p. GTT) at baseline (day 0) and day 7 of treatment. Asterisk (*) indicates *p* < 0.05 compared with WD on day 7, determined by mixed-model analysis. Exact *p* values as follows—WD + BAM15 0.05%, *p* = 0.0021; Chow and WD + BAM15 0.10% and WD + BAM15 0.15% *p* < 0.0001. Hash (#) indicates *p* = 0.0046 comparing baseline to final AUC by paired two-tailed Student’s *t* test. **g** Day 7 i.p.GTT curves. **h** Fasting glucose level recorded after a 5 h fast. Asterisk (*) indicates *p* < 0.05 compared with WD by one-way ANOVA. Exact *p* values—Chow, *p* = 0.046; WD + BAM15 0.10% and WD + BAM15 0.15%, *p* < 0.0001. **i** Serum insulin measured on treatment day 8. Asterisk (*) indicates *p* < 0.05 compared with WD by one-way ANOVA. Exact *p* values—Chow, *p* = 0.0112; WD + BAM15 0.10%, *p* = 0.0003; WD + BAM15 0.15%, *p* = 0.0005. All data presented as mean values ± SEM. Colour scale: green—Chow, red—Western diet (WD), light blue—WD + BAM15 0.05%, electric blue—WD + BAM15 0.10%, dark purple—WD + BAM15 0.15%. Source data provided in Source data file.

BAM15 admixed in WD at 0.1% w/w resulted in a drug exposure profile with desired bioactive effects observed for OCR and respiratory exchange ratio. Therefore, we next investigated the anti-obesity efficacy of BAM15. Male C57BL/6J mice were single housed to enable accurate food intake measurements and prevent variability related to fighting or huddling. Mice were fed WD or WD + BAM15 at 0.05%, 0.10%, and 0.15% w/w for 8 days. A separate group of age-matched chow-fed mice were used as controls for normal physiology. Body composition measured by serial EchoMRI every second day showed that BAM15 had a dose-dependent effect on fat mass such that 0.05% w/w BAM15 in WD prevented more than 50% of fat mass gain, while doses of 0.10–0.15% w/w BAM15 in WD completely prevented fat mass gain (Fig. 3a) without affecting fat-free lean mass or food intake (Fig. 3b, c). EchoMRI results were validated by daily body mass measurements (Fig. 3d) and dissection of four fat pad depots following euthanasia (Fig. 3e). Intraperitoneal glucose tolerance tests (i.p. GTT) were performed prior to treatment and on treatment day 7 (Fig. 3f). WD feeding for 7 days induced glucose intolerance in control mice while all three groups of mice fed BAM15 were protected from WD-induced glucose intolerance (Fig. 3f, g). The BAM15-induced improvements in glucose tolerance, compared with WD control mice, were associated with a dose-dependent decrease in fasting blood glucose (Fig. 3h) and decreased random-fed serum insulin levels (Fig. 3i). These data suggest that BAM15-induced improvements in glucose tolerance are likely related to improved insulin sensitivity.

### BAM15 reverses diet-induced adiposity

**Fig. 4:**
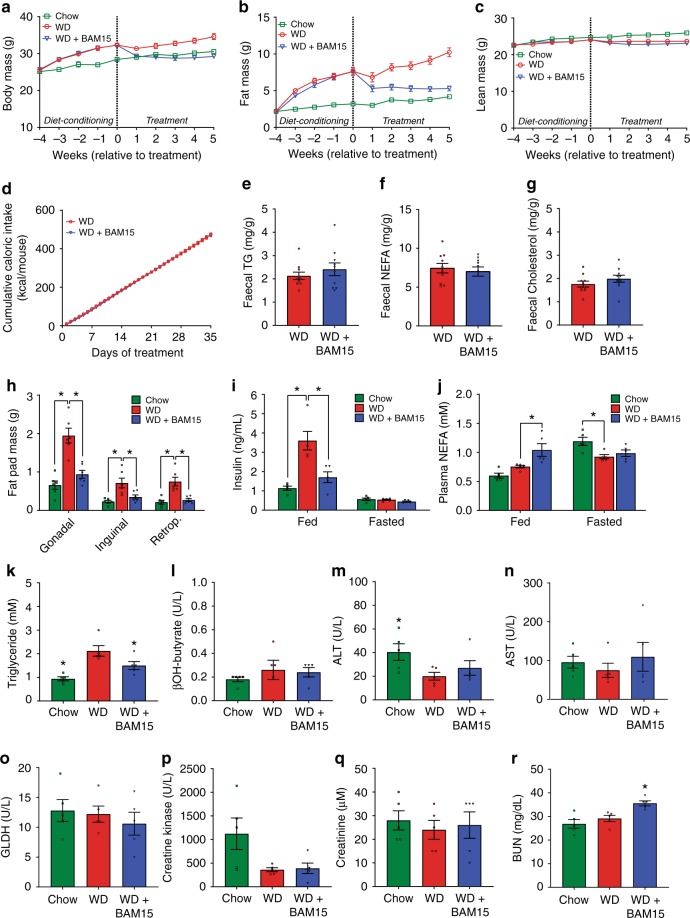
BAM15 reverses diet-induced adiposity without affecting lean mass or caloric intake. **a**–**d** Body mass, fat mass, lean mass, and cumulative food intake were recorded over the course of the study, *n* = 21 animals from four independent studies (except Week 1 fat mass and lean mass, *n* = 17 animals from three independent studies). **e**–**g** Fecal triglyceride, NEFA, and cholesterol levels comparing WD to WD + BAM15, *n* = 10 animals from two independent studies. Significance tested by two-tailed Student’s *t* test. **h** Final fad pad masses, *n* = 6 animals from one study. Asterisk (*) indicates *p* < 0.05 for each fat pad compared with WD, determined by one-way ANOVA. Exact *p* values as follows—Gonadal: Chow, *p* < 0.0001; WD + BAM15, *p* = 0.0003. Inguinal: Chow, *p* = 0.0015; WD + BAM15, *p* = 0.0125. Retroperitoneal: Chow, *p* = 0.0002; WD + BAM15, *p* = 0.0007. **i**, **j** Fed and 12 h fasted plasma insulin and NEFA concentrations, *n* = 5 animals from one study. Asterisk (*) indicates *p* < 0.05 for each treatment compared with WD, determined by two-way ANOVA. Exact *p* values as follows—Fed insulin; Chow and WD + BAM15, *p* < 0.0001. Fed NEFA; WD + BAM15, *p* = 0.0066. Fasted NEFA; Chow, *p* = 0.0118. Terminal plasma (**k**) triglyceride, (**l)** β-hydroxybutyrate, (**m**) ALT, (**n**) AST, (**o**) GLDH, (**p**) creatine kinase, (**q**) creatinine, and (**r**) BUN *n* = 5 animals from one study. Significance determined by one-way ANOVA, Asterisk (*) indicates *p* < 0.05 compared to WD. Exact *p* values as follows—Triglyceride: Chow, *p* = 0.0006; WD + BAM15, *p* = 0.0417. ALT: Chow, *p* = 0.0483. BUN: WD + BAM15, *p* = 0.0140. All data presented as mean values ± SEM. Colour scale: green—Chow, red—Western diet (WD), electric blue—WD + BAM15 0.10%. Source data provided in Source data file.

We next investigated whether BAM15 could reverse obesity and obesity-related metabolic disorders. Mice were fed WD for 4 weeks, which increased their body mass by >20% and increased fat mass by threefold relative to their pre-diet starting point (Fig. 4a, b, diet conditioning period). Mice were then single housed, stratified, and randomized to treatment groups of WD or WD containing 0.1% BAM15 w/w for a further 5 weeks. The 0.1% w/w concentration of BAM15 was chosen as it completely prevented diet-induced fat gain, had no effect on food intake, resulted in an average overnight drug exposure of 5–10 µM, and had beneficial metabolic effects in the obesity prevention study without affecting fat-free mass (Fig. 3). An age-matched chow-fed control group was used to benchmark normal physiologic phenotypes (Chow). At the end of the 9-week study the BAM15-treated group consumed the same calories as WD control mice but had 15% less body weight that consisted almost entirely of less fat mass with no difference in fat-free lean mass (Fig. 4a–d). Faecal triglyceride, NEFA and cholesterol content were not statistically different between the WD or WD + BAM15 fed groups (Fig. 4e–g) demonstrating that fat loss was not due to lipid malabsorption. BAM15 treatment significantly decreased fat pad masses (Fig. 4h), demonstrating that BAM15 treatment reversed diet-induced obesity.

At the mid-point of treatment (~2.5 weeks, days 18–19), insulin levels in WD + BAM15-fed animals were twofold less than WD controls and not statistically different from chow-fed mice (Fig. 4i). Plasma NEFA levels of BAM15-treated animals in the fed state were elevated by 40%, which is in the normal physiologic range observed during fasting (Fig. 4j).

Plasma biochemical and haematological parameters were assessed from blood collected at euthanasia (Supplementary Table 1 with key markers shown in Fig. 4k–r). Plasma triglyceride levels of mice fed WD + BAM15 were decreased by 29% compared with WD control mice (Fig. 4k). Despite increased fat oxidation, BAM15 treatment did not induce ketosis as β-hydroxybutyrate levels were similar to WD control mice (Fig. 4l). Indicators of liver, muscle, and heart tissue health including ALT, AST, GLDH, and creatine kinase were not affected by BAM15 treatment (Fig. 4m–p). Plasma creatinine and blood urea nitrogen (BUN) are typical measures of renal health but can also indicate alterations in protein metabolism. Compared with the WD controls, BAM15-treated mice had normal creatinine levels and mildly (22%) elevated BUN levels that remained within the normal reference range for male C57BL/6 J mice^32^ (Fig. 4q, r).

### BAM15 treatment improves liver antioxidant profile

**Fig. 5:**
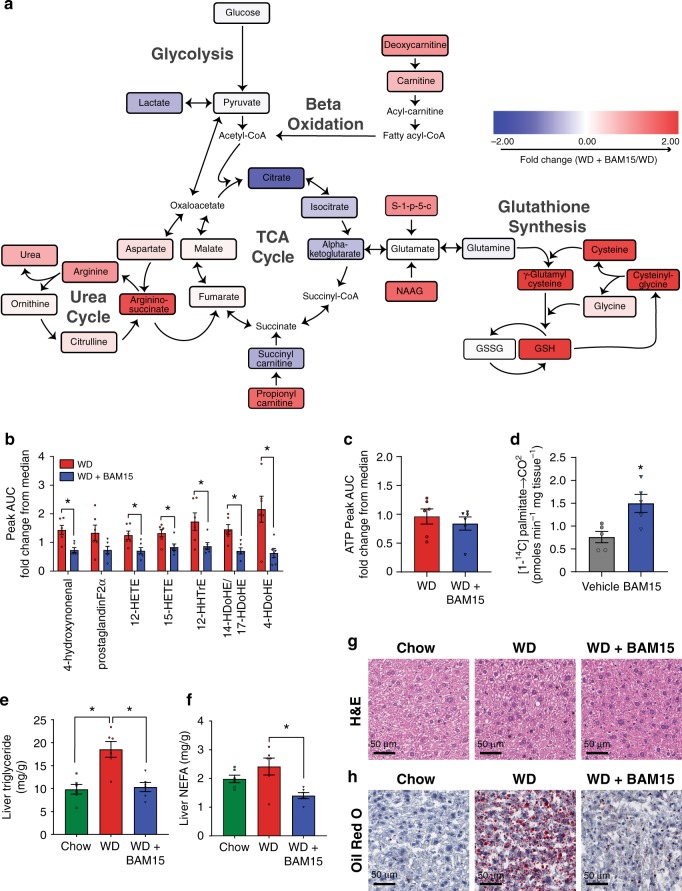
BAM15 increases hepatic lipid oxidation with minimal alteration to energy production pathways. Metabolomics analysis was performed on liver tissue. **a** Schematic showing key energy production pathways. Metabolites colour-coded by fold change of WD + BAM15 compared with WD, scale of twofold positive (red) to twofold negative (blue). **b** bioactive lipids and (**c**) ATP presented as fold change from median AUC for each metabolite (where median rescaled to equal 1), *n* = 6 animals from one study. Significance determined by two-tailed Student’s *t* test, asterisk (*) indicates *p* < 0.05, exact *p* values as follows; 4-hydroxynonenal, *p* = 0.0029, 12-HETE, *p* = 0.0138, 15-HETE, *p* = 0.0177, 12-HHTrE, *p* = 0.0256, 14-HDoHE/17-HDoHE, *p* = 0.0036, 4-HDoHE, *p* = 0.0097. **d** Complete fatty acid ([1-^14^C] palmitate) oxidation was measured in liver tissue collected 1 h post-oral gavage of BAM15 (100 mg/kg) or vehicle control, *n* = 5 animals form one study. Asterisk (*) indicates *p* < 0.05 determined by two-tailed Student’s *t* test, *p* = 0.0042. Liver tissue was analyzed for (**e**) triglyceride and (**f**) NEFA levels, asterisk (*) indicates *p* < 0.05 compared with WD by one-way ANOVA exact *p* values as follows—Liver triglyceride: Chow, *p* = 0.0005; WD + BAM15, *p* = 0.009. Liver NEFA; WD + BAM15, *p* = 0.0042. The same liver tissue was fixed and stained with (**g**) H&E and (**h**) Oil Red O, representative images shown, scale bar 50 µm. All data presented as mean values ± SEM. Colour scale: green—Chow, red—Western diet (WD), electric blue—BAM15 (WD + BAM15 0.10% or BAM15 100 mg/kg), grey—vehicle. Source data provided in Source data file.

BAM15 is primarily distributed to the liver; therefore, we investigated the effect of BAM15 on global metabolite changes in liver tissue following 20 days of treatment with 0.1% w/w BAM15 in WD. Compared to WD controls, 3% of liver metabolites (22 of 770 with a *p* value < 0.05) in WD + BAM15-fed animals were altered by more than twofold, and 0.1% of metabolites (1 of 770 with *p* < 0.05) were altered by more than fourfold (Supplementary Dataset 1). The metabolic pathways that were most altered are illustrated in Fig. 5a. Gamma-glutamylcysteine (GGC) was the metabolite with greatest statistically significant fold change (4.6-fold, *p* = 0.001). GGC is an important metabolic substrate for glutathione synthesis and antioxidant defence. Consistent with high levels of GGC, reduced glutathione (GSH) was increased 2.3-fold (*p* = 0.01) while oxidized glutathione concentrations (GSSG) were unaffected. Consistent with peptide antioxidant changes, the oxidised fatty acid 4-hydroxynonenal was also decreased by 49% (*p* = 0.002, Fig. 5b) in WD + BAM15-fed liver. The antioxidant profile of WD + BAM15 liver tissue was associated with lower levels of several bioactive pro-inflammatory eicosanoids and docosanoids (Fig. 5b). Together, these data indicate an antioxidant and anti-inflammatory phenotype in liver tissue of BAM15-treated mice.

TCA cycle intermediates citrate, isocitrate and α-ketoglutarate were not statistically altered with BAM15 treatment, but trended (*p* = 0.10–0.19) to be lower in abundance while the amino acid precursors of glutamate including 1-pyrroline-5-carboxylate (S-1-p-5-c) and N-acetyl-aspartyl-glutamate (NAAG) were increased by 46% and 69% (*p* < 0.05), respectively (Fig. 5a, Supplementary Dataset 1). Argininosuccinate, a precursor of fumarate and a urea cycle intermediate, was increased by 85% (*p* = 0.03) and liver urea levels were increased by 30% (*p* = 0.007) in BAM15-treated liver. Other amino acid metabolites including N-acetylserine, N-acetylthreonine, N-acetylleucine and N-acetylvaline were decreased (Supplementary Table 2). No significant changes in diacylglycerol or ceramide levels were observed compared to the WD control mice (Supplementary Table 3).

ATP levels were not altered in liver tissue of mice treated with BAM15 compared with WD control mice (Fig. 5c). Consistent with no change in ATP levels, we also did not detect a change AMPK phosphorylation or activity toward the AMPK substrate enzymes acetyl-CoA carboxylases in liver tissue of BAM15-treated mice (Supplementary Fig. 3).

BAM15 treatment decreased respiratory exchange ratio (RER) (Fig. 1f, i); therefore, we next assessed whether BAM15 had a direct effect on hepatic lipid oxidation by measuring ^14^C-palmitate oxidation to ^14^CO_2_ in liver tissue homogenates. Mouse liver tissue obtained 1 h following gavage with 100 mg/kg BAM15 had a 51% (*p* < 0.05) increase in palmitate oxidation compared with vehicle control (Fig. 5d). We next assessed hepatic lipid content in mice fed WD for 9 weeks with or without 0.1% w/w BAM15 treatment for the final 5 weeks. Compared with chow-fed mice, WD treatment doubled liver triglyceride levels while having little effect on intracellular NEFAs (Fig. 5e, f). BAM15 feeding resulted in complete correction of liver triglyceride content to levels similar to chow-fed mice and lowered NEFA levels by 42% relative to WD control. Consistent with these biochemical assay results, histological examination of liver sections revealed strong neutral lipid staining with Oil Red O in WD-fed animals that was corrected by BAM15 treatment (Fig. 5g, h).

### BAM15 reverses diet-induced insulin resistance

**Fig. 6:**
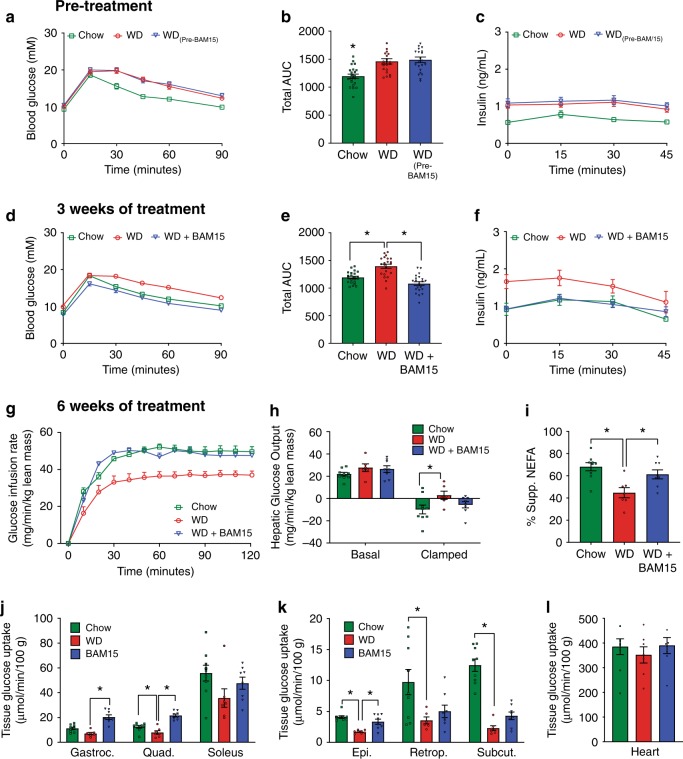
BAM15 reverses diet-induced insulin resistance. **a**–**c** Intraperitoneal glucose tolerance test (i.p.GTT) with insulin measurements performed on mice fed chow or WD for 4 weeks. **a**, **b** glucose measurements, *n* = 22 animals from four independent studies, significance tested with one-way ANOVA. Asterisk (*) indicates *p* < 0.05, exact value *p* < 0.0001. **c** Insulin measurements, *n* = 16 from three indepe*n*dent studies. **d**–**f** Mid-point i.p.GTT with insulin measurements conducted after 3 weeks of treatment. **d**, **e** glucose measurements, *n* = 22 animals from four independent studies, asterisk (*) indicates *p* < 0.05 determined by one-way ANOVA, *p* values for Chow and WD + BAM15 *p* < 0.0001. **f** Insulin measurements, *n* = 16 from three independent studies (except for 45 min timepoint, *n* = 5 from one study). **g**–**l** Hyperinsulinemic-euglycemic clamp parameters conducted after 6 weeks of treatment, *n* = 9 Chow, *n* = 7 WD, *n* = 8 WD + BAM15 animals from two independent studies; (**g**) glucose infusion rate, (**h**) suppression of hepatic glucose output, (**i**) suppression of non-esterified free fatty acids, and (**j**–**l**) tissue glucose uptake. Asterisk (*) indicates *p* < 0.05 compared with WD group by one-way ANOVA, except for (**h**) suppression of hepatic glucose output where significant difference compared with WD determined by two-way repeated measures ANOVA and *n* = 9 Chow, *n* = 6 WD, *n* = 8 WD + BAM15 animals from two independent studies. Exact *p* values as follows—(**h**) Clamped Chow, *p* = 0.0149. **i** Chow, *p* = 0.0011; WD, *p* = 0.0198. **j** Gastrocnemius (Gastroc.): BAM15, *p* < 0.0001; Quadricep (Quad.): Chow and BAM15, *p* < 0.0001. **k** Epididymal fat pad (Epi.): Chow and BAM15 *p* < 0.0001; Retroperitoneal fat pad (Retrop.): Chow, *p* = 0.0226; Subcutaneous fat pad (Subcut.): Chow, *p* < 0.0001. All data presented as mean values ± SEM. Colour scale: green—Chow, red—Western diet (WD), electric blue—BAM15 (WD + BAM15 0.10%). Source data provided in Source data file.

We next examined the effect of BAM15 on WD-induced insulin resistance. Glucose tolerance testing (Fig. 6a) confirmed that mice fed WD for 4 weeks were glucose intolerant and hyperinsulinemic at the end of the ‘conditioning phase’ prior to BAM15 intervention (Fig. 6b, c). This glucose intolerance and hyperinsulinemia were completely reversed by 3 weeks of treatment with 0.1% BAM15 in diet (Fig. 6d–f).

After 6 weeks of BAM15 treatment, insulin sensitivity was assessed by hyperinsulinemic-euglycemic clamp to determine which tissues contributed to the improved metabolic phenotype. The clamp data showed that BAM15 treatment markedly improved whole-body glucose clearance compared with WD control animals by increasing the glucose infusion rate required to maintain euglycemia to levels comparable with chow-fed mice (Fig. 6g and Supplementary Table 4). Insulin treatment in the clamped state partially suppressed hepatic glucose output in WD control mice and fully suppressed hepatic glucose output in chow-fed mice, while BAM15-treated animals had an intermediate phenotype that was not statistically different from either group (Fig. 6h). Mice fed WD + BAM15 had greater insulin-mediated suppression of adipose-derived NEFA production than WD controls (Fig. 6i). Compared with WD control mice, WD + BAM15-fed mice had greater [^14^C]2-deoxy-D-glucose uptake in mixed fibre type skeletal muscle (e.g. gastrocnemius and quadriceps) and epididymal adipose tissue (Fig. 6j, k). BAM15 treatment had no effect on heart glucose uptake (Fig. 6l).

## Discussion

Obesity is a complex disorder that is difficult to overcome. Obesity is influenced by genetic and environmental factors that include basal metabolic rate, satiety, hormone balance, physical activity, and food choices^2,33^. Weight loss is difficult to sustain in part because each person is individually programmed to defend a physiological set-point for body weight and initial weight loss is counteracted by feedback mechanisms that lower metabolic rate and/or increase hunger^34–37^. Calorie restriction is frequently used as an anti-obesity treatment, but it can have unwanted effects including depression and withdrawal symptoms particularly in patients with food addiction^35,38^. Importantly, interventions such as mitochondrial uncoupling can cause weight loss without affecting food intake. Pharmacologically decreasing metabolic efficiency by mitochondrial uncoupling represents a potential therapeutic intervention that may surmount genetic and environmental causes of obesity by overcoming the feedback mechanisms that defend body weight. For example, by definition, mitochondrial uncoupling causes metabolic inefficiency to directly override the body’s attempt to improve metabolic efficiency or lower metabolic rate. Mitochondrial uncoupling also does not cause complications associated with either undereating or overeating, indicating that it does not trigger the adaptive responses caused by calorie restriction or treatment with the SGLT2 inhibitor canagliflozin^37^. The lack of compensatory food intake in mice fed BAM15 is consistent with human data showing that people taking DNP did not report increased hunger^22^. The lack of altered food intake is psychologically important because the mesolimbic reward center in the brain is intimately connected with food consumption^39,40^; therefore, targeting metabolic efficiency with mitochondrial uncoupling may not be associated with the same psychological risk profile as dieting or using pharmacological agents that target satiety.

In this study, BAM15 demonstrated a direct on-target mechanism to lower metabolic efficiency that resulted in loss of body fat without altering activity or food intake. Mice fed BAM15 had lower RER values than control animals concomitant with elevated plasma NEFA levels suggesting that release of adipose-derived NEFAs fuel fatty acid oxidation in other tissues. We show direct evidence that BAM15 treatment increases oxygen consumption and ^14^C-palmitate oxidation in liver tissue ex vivo and decreases hepatic TG and NEFA in mice. These data demonstrate a direct effect of BAM15 to increase nutrient metabolism in the liver with lesser effects evidenced in skeletal muscle and fat. BAM15 treatment had no effect on fat-free lean mass, no impact on body temperature, and no effect on clinical biochemistry or haematology indicators of tissue damage. These rigorous assessments show that BAM15 safely reversed obesity and insulin resistance without observed adverse effects. The only serum parameters that differed statistically after chronic BAM15 treatment were lower triglyceride and higher BUN levels. The average BUN level in WD + BAM15-fed mice was 35 mg/dL, which is not considered elevated as it is within the normal physiological range for C57BL/6 mice^41^. Furthermore, creatinine levels were normal and acute oral gavage of BAM15 did not alter BUN levels.

Metabolomic analysis of liver tissue from WD + BAM15-fed mice showed that there was no major destabilisation across the metabolic profile as few metabolites changed more than twofold. The strongest signatures were antioxidant effects, evidenced by an increased GSH:GSSG ratio, decreased bioactive oxidised lipids (e.g. 4-HNE), and decreased pro-inflammatory lipid mediators in the form of eicosanoids and docosanoids. The antioxidant effect was expected because mild mitochondrial uncoupling has antioxidant effects that decrease superoxide production from the electron transport chain^42,43^. Metabolomics data identified changes in amino acid metabolism that may be related to increased amino acid oxidation or anaplerosis into the TCA cycle. Increased anaplerosis is an expected phenotype because it counterbalances the increase in fat oxidation to prevent outstripping the TCA cycle^44^. Mice treated with BAM15 had normal ATP levels and there was no indication of energy stress via AMPK signaling compared to control mice, thereby indicating that nutrient flux through mitochondria in BAM15-treated liver was sufficient to maintain normal physiology without energetic stress.

In the WD reversal study, mice fed WD for 4 weeks had a threefold increase in fat mass. Groups of mice were stratified and randomized to WD with or without 0.1% w/w BAM15. Within 3 weeks of treatment, BAM15 completely reversed diet-induced glucose intolerance and hyperinsulinemia. Hyperinsulinemic-euglycemic clamp experiments performed after 6 weeks of treatment showed that BAM15 improved insulin sensitivity to levels similar to chow mice evidenced by normalization of glucose infusion rate and improvements in muscle glucose clearance and suppression of adipose NEFA production. WD-fed mice have impaired insulin-mediated suppression of hepatic glucose output compared to chow-fed mice; however, mice fed WD containing BAM15 have an intermediate phenotype between chow and WD whereby they are statistically no different from either chow or WD groups. When considering this collectively with the biochemical data, BAM15 appears to have a stronger phenotype for fat oxidation over glucose metabolism that may underlie the anti-obesity effects. Cardiac muscle glucose uptake was not statistically different for any group.

Recent years have seen a resurgence of interest in mitochondrial uncouplers as human medicines. For example, the FDA recently granted IND approval for DNP to re-enter the clinic as a potential therapeutic for patients with Huntington’s disease^45^, and niclosamide and nitazoxanide are FDA-approved anti-parasitic drugs that were discovered to have mitochondrial uncoupling activity. Nitazoxanide is not known to have anti-obesity effects, but it is currently in phase 2 clinical trials for NASH-induced fibrosis^46^. Niclosamide has been studied in obese mouse models where it slowed not only fat mass gain but also decreased lean mass gain when administered in a diet-induced obesity prevention model^47^. Niclosamide unexpectedly increased body weight when fed to *db*/*db* mice^47^. In contrast to niclosamide, BAM15 treatment in the obesity prevention model decreased fat mass below that of normal chow-fed mice without decreasing lean mass thereby revealing a remarkably high specificity for BAM15 to target fat loss. The preservation of lean mass during weight loss is highly desired and distinguishes BAM15 from other uncouplers. Another mitochondrial uncoupler OPC-163493 recently discovered by Otsuka Pharmaceuticals had strong anti-diabetes effects in multiple rodent models, but did not impact adiposity^29^. Finally, controlled-release DNP and the methyl ether prodrug version of DNP both failed to alter adiposity^25,26^.

In summary, BAM15 represents a rare mitochondrial uncoupler that prevents and reverses obesity without affecting food intake or lean mass. One limitation of BAM15 is low aqueous solubility, but this property did not affect oral bioavailability and indeed low aqueous solubility is an important parameter that enables BAM15 to penetrate membranes and enter mitochondria. Another limitation of BAM15 is a 1.7 h half-life and future directions will investigate formulation strategies to improve exposure. Collectively, the data presented herein supports further development of BAM15 as a potential therapeutic for obesity and metabolic diseases.

## Methods

### Study design

The primary objective of this research was to assess the anti-obesity efficacy of BAM15 in diet-induced adiposity. Sample size estimates were gated on the number of animals needed to observe a 50% decrease in weight gain with a standard deviation of 10–15% of the mean, in both obesity prevention and reversal studies. Using calculations with alpha 0.05 and power 80%, the required sample size was calculated to be six mice per group. Sample sizes of >6 were used for most experiments. In two cases data from the hyperinsulinemia-euglycemic clamp study was excluded for technical reasons including an animal that did not recover from catheter surgery prior to hyperinsulinemia-euglycemic clamp and an animal that had a catheter become loose during the clamp experiment. GTT data from one animal at one timepoint was excluded due to a mis-injection of glucose. No animals were excluded in other experiments, and no experiments were terminated prematurely. Mice were stratified to ensure body composition and baseline glucose tolerance were matched across treatment groups then randomly assigned to treatment groups.

### Oxygen consumption rate Seahorse assay

OCR was measured using an Agilent Seahorse XFe96 Analyzer (Agilent Technologies, Santa Clara, CA). NMuLi cells (American Type Culture Collection, Manassas, VA) were seeded in a 96-well seahorse plates at a density of 2.0 × 10^4^ cells/well and were allowed to adhere overnight. Prior to the assay, media was changed to unbuffered DMEM containing pyruvate and glutamine (Gibco 12800-017) pH = 7.4 at 37 °C and the cells were equilibrated for 1 h at 37 °C without CO_2_. Compounds were injected during the assay and OCR was measured using 2 min measurement periods. Cells were treated with a single drug concentration. Three independent experiments were conducted with two to four replicates per treatment condition per experiment.

### Animal diets

WD (45% fat and 16% sucrose by kCal, based on Research Diets D12451) was prepared in-house as described in Healy et al.^48^. Ingredients were sourced from local suppliers including sucrose (JL Stewart, GRAD25B, Australia), corn starch (JL Stewart, CFLR25W, Australia), wheat bran (JL Stewart, BRAN10UF, Australia), casein (Ross Cootee, Australia), methionine (Sigma, M9500, Australia), gelatine (JL Stewart, GEL2, Australia), choline bitartrate (Sigma, C1629, Australia), lard (JL Stewart, LARD15, Australia), safflower oil (Protec, Australia), trace minerals (MP Biomedicals, 0296026401, Australia), AIN-93M mineral mix (MP Biomedicals, 0296040102, Australia), and AIN-93-VX vitamin mix (MP Biomedicals, 0296040201, Australia).

### Phenotyping studies

All mouse experiments complied with all relevant ethical standards regarding animal research. All mouse experiments were approved by Animal Care and Ethics Committees at the UNSW (project 14-33A, 20/67A, 17-66B and 18/91A) and Garvan Institute (project 18/19). Age-matched male C57BL/6J mice were used in the studies as indicated. Mice were purchased from Australian BioResources (Moss Vale, NSW, Australia). Mice were housed at 22 °C in a light/dark cycle of 12 h. Unless otherwise stated, mice were provided with ad libitum access to water and standard chow diet (Gordons Specialty Feeds, NSW, Australia).

For the obesity-prevention dose response study, we first stratified a cohort of 4-month-old male C57BL/6J chow-fed mice by baseline body composition and glucose tolerance to ensure similar starting parameters. EchoMRI was used to establish body fat and lean mass. Glucose tolerance was assessed by intraperitoneal injection of 2 g glucose per kg lean body mass following a 5 h fast from 9 a.m. to 2 p.m. Mice were single-housed to ensure accurate monitoring of food intake prior to changing diet to WD without or with BAM15 at 0.05, 0.1, and 0.15% w/w for 8 days. EchoMRI was used to measure changes in fat and lean mass over 7 days and a glucose tolerance test was repeated on day 7. Mice were euthanised by cervical dislocation and harvested tissues were frozen in liquid nitrogen prior to storage at −80 °C.

For the obesity-reversal study, 2-month-old male C57BL/6J mice were fed either chow diet or WD for 4 weeks. All mice were assessed for body composition on a weekly basis by EchoMRI, and glucose tolerance testing was performed on week 4 prior to single housing and stratification into two groups. One group of mice continued to be fed WD while the other group was fed WD containing 0.1% (w/w) BAM15. Food intake and body weight were measured daily. On the final day of study mice were anesthetised with isoflurane and exsanguinated by retroorbital bleeding for clinical biochemistry and hematology.

Indirect calorimetry was performed using an Oxymax CLAMS (Columbus Instruments’ Comprehensive Lab Animal Monitoring System, USA) indirect calorimeter in the Biological Resource Centre at UNSW. The flow rate of air was 0.49 L/min and concentration of incoming and outgoing O_2_ and CO_2_ were measured to calculate O_2_ consumption, CO_2_ production and respiratory exchange ratio (*V*CO_2_/*V*O_2_).

Core body temperature was measured with a rectal probe thermometer (Braintree, TW2-107). Maximal rectal temperature that resulted in a stable reading for >5 s was recorded.

### BAM15 exposure

Pharmacokinetic assessments were conducted on 2-month-old male C57BL/6J mice. Mice were administered 1 mg/kg BAM15 intravenously (in NMP (Sigma, 328634) (10% v/v) + 10% Kolliphor^®^ EL (Sigma, C5135) (v/v), in water) or 10 mg/kg by oral gavage (0.7% w/v methylcellulose (Sigma, M0512) (93% v/v), 2% v/v Tween-80 (Sigma, P6474), and 5% v/v DMSO (Sigma, D5879). Blood samples were collected in heparinized capillary collection tubes (Sarstedt, 20.1309, Germany) at time points indicated in the figure. Standards were prepared by spiking known concentrations (0.1, 1, 10 and 100 ng) of BAM15 into vehicle treated whole blood prior to extraction. Plasma was separated by centrifugation (2000 × *g* for 10 min). 7 μL plasma was precipitated in 100 μL of 90% (v/v) acetonitrile and 10% (v/v) methanol. The solution was briefly vortexed then centrifuged (18,000 × *g* for 10 min). Supernatant was collected in auto-sampler vials for mass spectrometry.

Liquid chromatography tandem mass spectrometry was performed on a Shimadzu Prominence LCMS-8030 (Shimadzu, Japan). Chromatographic separation was achieved using an ACUITY UPLC BEH, C18 column (Waters, WT186002350, USA). Mobile phase A consisted of 0.1% v/v formic acid in HLPC-grade water. Mobile phase B consisted of 0.1% v/v formic acid in acetonitrile. The analyte was eluted with a gradient of 5–80% mobile phase B at a flow rate of 0.4 mL/min with 10 μL injection volume electrosprayed into the mass spectrometer. ESI was performed in positive mode. Transition of *m/z* 341 > 162, and 6 min retention time was used to identify BAM15. Quantification was determined by measuring peak areas using LabSolutions Software on the instrument. Concentrations of test samples were interpolated from a standard curve derived from the intensity values of standards. Quantified values were normalised to tissue weight.

For tissue distribution studies 2-month-old male C57BL/6J mice were treated with 50 mg/kg BAM15 by oral gavage. Mice were euthanised by cervical dislocation after 0.5, 1, 2, and 4 h. Tissues were dissected, rinsed in PBS or blotted to remove blood and immediately frozen in liquid nitrogen. Frozen tissues were powdered in a tissue pulveriser (Cellcrusher, USA) cooled by liquid nitrogen. Powdered tissue samples were homogenised in PBS using a motorised pellet pestle homogeniser. Homogenate was centrifuged (1000 × *g* for 10 min) and supernatant collected. BAM15 was extracted by adding supernatant (1:9) to a solution of 90% (v/v) acetonitrile and 10% (v/v) methanol. The solution was briefly vortexed then centrifuged (18,000 × *g* for 10 min). Supernatant was collected in auto-sampler vials for mass spectrometry.

The overnight drug exposure study involved blood collection from the tail tip in heparinised capillary collection tubes (Sarstedt, 20.1309, Germany) and stored on ice prior to centrifugation to separate plasma (2000 × *g* for 10 min).

### High-resolution respirometry

High-resolution respirometry was conducted in C57BL/6J mouse tissue as per Canto and Garcia-Roves^49,50^, using the OROBOROS Oxygraph-O2K (Oroboros Instruments, Corp., Innsbruck, AT). Briefly, mice were treated with and oral gavage of BAM15 at 50 mg/kg or a vehicle control. One-hour post-treatment mice were anesthetized using isoflurane gas, and quadricep muscle (quad), right lobe liver (liver), gonadal white adipose tissue (gWAT) and brown adipose tissue (BAT) were dissected and placed in BIOPS preservation solution (50 mM K^+^-MES, 20 mM taurine, 0.5 mM dithiothreitol, 6.56 mM MgCl_2_, 5.77 mM ATP, 15 mM phosphocreatine, 20 mM imidazole pH 7.1 adjusted with 5 N KOH at 0 °C, 10 mM Ca-EGTA buffer (2.77 mM CaK_2_EGTA + 7.23 mM K_2_EGTA; 0.1 μM free calcium^50^. Prior to addition of the samples to the OROBOROS chamber, tissues were prepared in MiR05 buffer (110 mM sucrose, 60 mM K^+^-lactobionate, 0.5 mM EGTA, 3 mM MgCl2, 20 mM taurine, 10 mM KH_2_PO_4_, 20 mM HEPES, 1 g L − 1 BSA essentially fatty acid-free, pH 7.1 with KOH at 37 °C). The OROBOROS chambers contained 2 mL MiR05 with blebbistatin (25 µM), stirred at a constant speed of 12.5 Hz (750 rpm) at 37 °C, and oxygen levels were maintained between 150 and 220 µM. gWAT was added to the OROBOROS chamber in small 20 mg pieces to a total concentration of 30–40 mg/mL. Liver and BAT was homogenized in ice cold MiR05 buffer at a concentration of 1 mg/10 µL using a pestle homogenizer, and 2 mg (20 µL) of tissue was added to the respiratory chamber. For skeletal muscle, muscle fibres were separated into small bundles, and permeabilized for 30 min at 4 °C in BIOPS buffer with 50 µg/ml saponin. Permeabilized muscle fibre bundles were washed in MiR05, before being added to the OROBOROS chamber at a final concentration of 1.25–5 mg/mL. Pyruvate (10 mM) and malate (2 mM) were added to the closed chambers using a Hamilton syringe and O_2_ concentration, CO_2_, and O_2_ consumption by the biological sample (JO_2_) were recorded.

### Blood collection and analysis

Fed blood was collected from a tail nick in heparinised capillary collection tubes (Sarstedt; 20.1309, Germany) coated tubes between 9 and 10 p.m. Food was then removed overnight, and fasted bloods were collected at 9–10 a.m. the following morning. Blood samples were centrifuged at room temperature, 2000 × *g* and plasma was frozen for later analysis. Insulin concentrations were assayed using Crystal Chem Ultra-Sensitive Mouse Insulin ELISA Kit (Crystal Chem Inc., 90080, USA) according to manufacturers’ instructions except that test samples and standards were incubated overnight at 4 °C.

Terminal blood draws were collected under isoflurane anaesthesia via retroorbital bleed in ETDA-coated capillary tubes (Sarstedt; 15.1671.100, Germany). Hematology and clinical biochemistry panels were performed by Laverty Pathology Vetnostics (West Ryde, NSW, Australia). Haematological analysis was performed using a Sysmex XN-V Multisciences hematology analyser (Sysmex, USA). Plasma was then extracted by centrifugation and clinical biochemistry panel was performed using a Cobas 8000 modular analyser (Roche, USA).

### Lipid analysis

Fecal samples were collected at 7:30–8:00 a.m. and stored at −80 °C. Liver tissues were snap-frozen in liquid nitrogen and stored at −80 °C until lipid extraction. Lipids were extracted from powdered liver tissue or faecal pellets using a modified version of Folch et al.^51^ method. In brief, 800 μL 2:1 chloroform-methanol (v/v) was added to ~25 mg of powdered liver or a single faecal pellet. The tissue was homogenised with a motorised pellet pestle, followed by 10 pulses with a probe sonicator, then vortex mixed for 20 s. Samples were digested for 1 h at room temperature on a rocker. Four hundred microlitres 0.6% NaCl was added to each sample and then vortex mixed. Samples were centrifuged (3000 × *g*, 10 min) and the bottom phase (lipid extract) was collected. Centrifugation and collection of the lipid extract was repeated once. The lipid extract was dried under a steady stream of nitrogen in a TurboVap^®^ Evaporator (Biotage, USA). The extract was resuspended in 0.4 mL 95% ethanol and heated to 37 °C prior to lipid assays. Colorimetric assays were used to measure triglycerides (Pointe Scientific, T7532) and cholesterol (Thermo Scientific, TR13421), and fluorometric assay used to measure NEFA (Wako Chemical, 279-75401). All assays were conducted according to the manufacturer’s protocols. Results were normalised to mass of liver tissue or faecal sample.

### H&E stained liver

Liver tissue was fixed in 10% neutral buffered formalin for 24 h (Sigma Aldrich, HT501128). Tissue was rinsed in PBS then stored in 70% ethanol. Fixed tissue was embedded in paraffin, sectioned 4 µm thick, and stained with Hematoxylin instant and Eosin-Y solution with phloxine (ThermoFisher Scientific) by the Histopathology Department at the Garvan Institute of Medical Research (Sydney, NSW, Australia).

### Oil Red O stained liver

Liver samples were collected, placed in cryomolds (Tissue Tek, 4566-100), covered with O.C.T.™ compound (Tissue Tek, 4583) and frozen on dry ice. Frozen tissue sections were cut 7 µm thick and mounted on slides by the Garvan Institute of Medical Research Histopathology Department. Oil Red O staining was performed by the Histology Core at UNSW. Slides were air dried for 30–60 min at room temperature and then fixed in ice cold 10% formalin for 10 min. Fixed slides were air dried and then rinsed in distilled water three times. Slides were placed in absolute propylene glycol for 5 min, then stained in solution of pre-warmed 0.5% Oil Red O in propylene glycol for 8–10 min in a 60 °C oven. The stain was differentiated in 85% propylene glycol solution for 5 min, then rinsed twice with distilled water. Slides were placed in Harris haematoxylin for 30 s then washed thoroughly in running tap water for 3 min. Slides were placed in distilled water and mounted in glycerine jelly.

### Lipidomic and metabolomic analyses

Frozen liver samples collected from mice euthanized in the random-fed state on day 20 of BAM15 or control treatment were sent to Metabolon Inc. for metabolomic analysis (North Carolina, USA). Samples were prepared using the automated MicroLab STAR^®^ system from Hamilton Company. Proteins were precipitated with methanol under vigorous shaking for 2 min (Glen Mills GenoGrinder 2000) followed by centrifugation. The resulting extract was divided into five fractions: two for analysis by two separate reverse phase (RP)/UPLC-MS/MS methods with positive ion mode electrospray ionization (ESI), one for analysis by RP/UPLC-MS/MS with negative ion mode ESI, one for analysis by HILIC/UPLC-MS/MS with negative ion mode ESI, and one sample was reserved for backup. Samples were placed briefly on a TurboVap^®^ (Zymark) to remove the organic solvent. The sample extracts were stored overnight under nitrogen before preparation for analysis. All methods utilized a Waters ACQUITY ultra-performance liquid chromatography (UPLC) and a Thermo Scientific Q-Exactive high-resolution/accurate mass spectrometer interfaced with a heated electrospray ionization (HESI-II) source and Orbitrap mass analyzer operated at 35,000 mass resolution.

The sample extract was dried then reconstituted in solvents compatible to each of the four methods. One aliquot was analyzed using acidic positive ion conditions, chromatographically optimized for more hydrophilic compounds. In this method, the extract was gradient eluted from a C18 column (Waters UPLC BEH C18-2.1 × 100 mm, 1.7 µm) using water and methanol, containing 0.05% perfluoropentanoic acid (PFPA) and 0.1% formic acid. Another aliquot was also analyzed using acidic positive ion conditions; however, it was chromatographically optimized for more hydrophobic compounds. In this method, the extract was gradient eluted from the afore-mentioned C18 column using methanol, acetonitrile, water, 0.05% PFPA and 0.01% formic acid and was operated at an overall higher organic content. Another aliquot was analyzed using basic negative ion optimized conditions using a separate dedicated C18 column. The basic extracts were gradient eluted from the column using methanol and water, however with 6.5 mM ammonium bicarbonate at pH 8. The fourth aliquot was analyzed via negative ionization following elution from a HILIC column (Waters UPLC BEH Amide 2.1 × 150 mm, 1.7 µm) using a gradient consisting of water and acetonitrile with 10 mM ammonium formate, pH 10.8. The MS analysis alternated between MS and data-dependent MSn scans using dynamic exclusion. The scan range varied slighted between methods but covered 70–1000 *m*/*z*.

Raw data was extracted, peak-identified and QC processed using Metabolon’s hardware and software. Peak AUC for each metabolite was log_2_ transformed for subsequent analysis.

### Hepatic lipid oxidation assay

Assessment of hepatic lipid oxidation was performed using a protocol derived from Yu et al.^52^. In summary, 5-month-old male C57BL6/J mice were treated by oral gavage of 100 mg/kg BAM15 or vehicle control. One-hour post-gavage mice were euthanized by cervical dislocation and whole livers were removed and immediately placed in ice-cold isolation buffer (220 mM mannitol, 70 mM sucrose, 2 mM HEPES, 0.1 mM EDTA, in milliQ water (pH 7.4)). A small portion of liver was blotted to remove excess liquid and weighed. A liver section (~100 mg) was placed in 100 µL ice-cold incubation buffer (isolation buffer containing 1.33 mM L-carnitine, 0.133 mM coenzyme A, 1.33 mM malic acid in milliQ water (pH 7.4)) and minced with scissors. Minced tissue was further diluted in incubation buffer (50 mg tissue/mL incubation buffer) and homogenised using six strokes of a loose fitting dounce homogenizer. Samples were kept on ice before transferring liver homogenate to a 24-well plate (100 µL/well, 3–6 wells per liver). A pre-warmed solution containing 1% fatty acid-free BSA-conjugated palmitate was spiked into each well (33 µL/well). The final concentration of palmitate was 125 µM with 2 µCi/mL 1-^14^C-palmitate (NEC075H050UC, Pekin Elmer, Australia), and the final concentrations of L-carnitine, coenzyme A and malic acid were 1, 0.1 and 1 mM, respectively. Traps containing NaOH were added into each well, wells were sealed, and plates incubated for 20 min at 37 °C. The reaction was stopped by injecting 100 µL of 2M perchloric acid per well. Wells were resealed and plates were incubated at room temperature for 1–2 h. Evolved ^14^CO_2_ was measured by scintillation counting. Wells containing incubation buffer and palmitate, but without liver homogenate, were used as background controls. Activity of samples was calculated relative to the specific activity of 1-^14^C-palmitate solution. Disintegrations per minute counts from background controls were subtracted from all liver homogenate counts and the data normalised to mass of liver tissue.

### Hyperinsulinemic-euglycemic clamp study

The hyperinsulinemic-euglycemic clamp study was performed at the end of the 6-week BAM15 treatment study. Mice were implanted with dual jugular and carotid catheters as described by Brandon et al.^53^, 1 week prior to the clamp study. The hyperinsulinemic-euglycemic clamp was conducted following a 5 h fast. Mice were conscious, unrestrained and were not handled during the procedure to minimise stress. A primed (5 μCi) continuous infusion (0.05 μCi/min) of [3,^3^H]-glucose (PerkinElmer, Victoria, Australia) was commenced for 90 min. At times 60, 70, 80, and 90 min blood samples were collected for basal glucose turnover (Rd). Following 90 min of baseline readings the rate of [3,^3^H]-glucose was increased to (0.1 μCi/min) and a primed (24 μU/kg) continuous (6 μU/kg per min) infusion of insulin was commenced (Actrapid, Novo Nordisk, Copenhagen, Denmark). Glucose (25%) was infused at a variable rate to maintain glycaemia at ~8 mM. Once blood glucose was stable, four sequential samples were taken to determine glucose turnover. A bolus of 2[^14^C]-deoxyglucose (10 μCi; PerkinElmer) was then administered and blood sampled at 2, 5, 10, 20, and 30 min. Animals were then euthanised and organs removed, freeze clamped and frozen in liquid nitrogen, then stored at −80 °C until analysis. This protocol is based on Ayala et al.^54^ and Charbonneau and Marette^55^. The rate of glucose disappearance (Rd) and tissue glucose uptake were determined as described by Brandon et al.^53^.

### Statistics and reproducibility

All data points were collected from discrete samples and are presented as the mean ± S.E.M. Significance was determined to be reached when *p* < 0.05 using R (v.3.5.3) or Prism (v.8.1.2; GraphPad Software). When data were normally distributed differences between groups were examined using the following tests: unpaired two-tailed Student’s *t* test where one group was compared to control; paired two-tailed Student’s *t* tests for comparing baseline to final treatments, one-way analysis of variance (ANOVA) for comparison of more than two groups, and two-way ANOVA for comparison of more than two groups with repeated measures, or where values were missing from repeated measures, mix model analysis was used. Two-tailed unpaired *t* test with Welch’s correction was used for data sets with unequal variance. Dunnett’s post-hoc test was used for all multiple comparisons, unless otherwise specified.

Reversal and prevention studies with 0.01% BAM15 in WD were replicated in several cohorts that are combined. Figure legends identify the number of studies combined and reported in each figure. Overall, the prevention study has been repeated three times with similar results. The reversal study has been repeated six times, for different lengths of study duration, with similar results. The Oxymax CLAMS experiments and the tolerability studies were performed once with at least four mice per group.

### Reporting summary

Further information on research design is available in the Nature Research Reporting Summary linked to this article.

## Supplementary information

Supplementary Information

Description of Additional Supplementary Files

Supplementary Dataset 1

Reporting Summary

## Data Availability

The authors declare that all the data supporting the findings of this study are available within the paper, the supplementary figures or the source data provided. The Source data underlying Figs. 1b–i, 2a, d, g–i, 3a–i, 4a–r, 5b–f, 6a–l, Supplementary Figs. 1–3 and Supplementary Tables 1–3 are provided as a Source Data file.

## Source Data

Source Data
