# Supplementary material for: Mitochondrial uncoupler BAM15 reverses diet-induced obesity and insulin resistance in mice
Source: Nat Commun. 2020 May 14;11:2397. doi: 10.1038/s41467-020-16298-2 (PMC7224297; doi:10.1038/s41467-020-16298-2)
Supplement: Supplementary file 1 — Supplementary Information [file 41467_2020_16298_MOESM1_ESM.pdf]

Supporting Information

Mitochondrial uncoupler BAM15 reverses diet-induced obesity and insulin resistance in mice

Stephanie J. Alexopoulos[1], Sing-Young Chen[1], Amanda E. Brandon[2], Joseph M. Salamoun[3,] Frances L. Byrne[1], Christopher J. Garcia[3], Martina Beretta[1], Ellen M. Olzomer[1], Divya P. Shah[1], Ashleigh M. Philp[4], Stefan R. Hargett[5], Robert T. Lawrence[5], Brendan Lee[6], James Sligar[4], Pascal Carrive[7], Simon P. Tucker[8], Andrew Philp[4], Carolin Lackner[9], Nigel Turner[10], Gregory J. Cooney [2], Webster L. Santos[3,8]*, and Kyle L. Hoehn[1,5,8]*

1. School of Biotechnology and Biomolecular Sciences, University of New South Wales, Sydney, NSW, 2052, Australia.
2. Sydney Medical School, Charles Perkins Centre, University of Sydney, Sydney, NSW, 2006, Australia
3. Department of Chemistry and Virginia Tech Centre for Drug Discovery, Virginia Tech, Blacksburg, VA, 24061, USA.
4. Garvan Institute of Medical Research, Darlinghurst, NSW, Australia.
5. Department of Pharmacology, University of Virginia, Charlottesville, Virginia 22908.
6. Biological Resources Imaging Laboratory, University of New South Wales, Sydney, NSW, 2052, Australia.
7. Department of Anatomy, School of Medical Sciences, University of New South Wales, Sydney, NSW, 2052, Australia.
8. Continuum Biosciences Pty Ltd. Sydney, NSW, 2035, Australia.
9. Institute of Pathology, Medical University of Graz, Graz, Austria.
10. Department of Pharmacology, School of Medical Science, University of New South Wales, Sydney, NSW, 2052, Australia.

*corresponding authors: k.hoehn@unsw.edu.au for biology and santosw@vt.edu for chemistry
Keywords: Metabolism; physiology; obesity

Supplemental Methods: Chemistry

BAM15 synthesis and purification

BAM15 (Figure 1a) was synthesised from diaminofurazan via a three-step procedure[1-3]

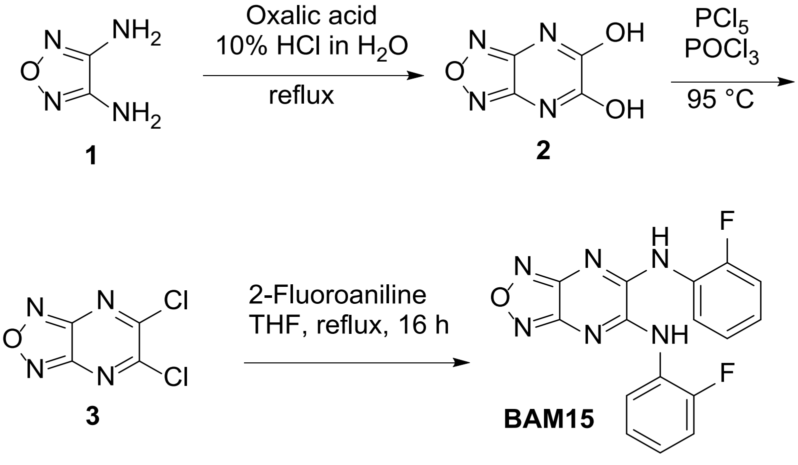

Synthesis of [1,2,5]oxadiazolo[3,4-*b*]pyrazine-5,6-diol **2**.

In a 3 L, 3-neck round-bottom flask equipped with a condenser, an internal thermometer, and a glass stopper, a mixture of 1,2,5-oxadiazole-3,4-diamine (**1**) (300 g, 3.00 mol) and oxalic acid (300 g, 3.33 mmol) was diluted with aq. HCl (1.6 L, 10% v/v). The resulting thick mixture was heated to reflux (ca. 100 °C). After 5 hours, the solution was transferred to a large beaker and allowed to cool to room temperature while a thick precipitate formed. The precipitate was filtered, rinsed with minimal amount of water (ca. 100 mL), and dried in a vacuum oven at 40-50 °C with P_2O_5 as a desiccant to yield dihydroxy **2** (279 g, 60%) as an off-white solid: ^{1}H NMR (($CD_3)_2CO$, 400 MHz) δ 11.86 (bs, 2H); ^{13}C NMR (($CD_3)_2CO$, 100 MHz) δ 154.0, 144.7.

Synthesis of 5,6-dichloro[1,2,5]oxadiazolo[3,4-*b*]pyrazine **3**.

In a 1 L three-neck round-bottom flask equipped with two glass stoppers and a condenser connected to an aq. Na_2CO_3 trap, $POCl_3$ (100 mL) was added to a mixture of dihydroxy **2** (75.0 g, 487 mmol) and PCl_5 (227 g, 1.09 mol) and the resulting mixture was slowly heated to reflux (ca. 105 °C) for 1.5 h, turning to a dark yellow solution, until bubbling stopped. The first major wave of bubbling began at around 35 °C and then a second wave at ca. 95 °C. The mixture cooled to room temperature and then slowly quenched by pouring into room temperature water (1 L in 2 L flask), not allowing the temperature of the water to rise beyond 50 °C (controlled by the addition of ice). The colorless precipitate was filtered, washed with water, and dissolved in acetone (ca. 500 mL). Water (3x volume of acetone) was added to the acetone solution to promote precipitation. The precipitate was filtered, rinsed with water, collected, and dried in a vacuum oven at 40-50 °C with P_2O_5 as a desiccant to yield dichloro **3** (56 g, 61%) as a colorless powder. ^{13}C NMR (($CD_3)_2CO$, 100 MHz) δ 156.0, 152.0. Note: DMSO-d_6 should not be used as a solvent as it will hydrolyze the product back to starting material. Analytical data is consistent with literature[4].

Synthesis of N^5,N^6-*bis*(2-fluorophenyl)[1,2,5]oxadiazolo[3,4-*b*]pyrazine-5,6-diamine **BAM15**.

In a 1 L flask equipped with a condenser, 2-fluoroaniline (70 mL, 0.73 mol) was added portion-wise over 10 minutes to a mixture of dichloro **3** (33.0 g, 0.173 mol) in anhydrous THF (500 mL). The resulting mixture was stirred at 70 °C for 16 h, allowed to cool to rt, and filtered to remove the salts. The filtrate was cooled in an ice bath for ca. 1 h to promote precipitation of a yellow solid. The solid was filtered, rinsed with a small amount of MeOH (ca. 20-30 mL). The filtration and rinsing procedure were repeated on the filtrate until all of the desired product was recovered as a bright yellow solid. Then, the solid was dissolved in a minimal amount of hot acetone (forms a clear light orange solution), which was slowly cooled to room temperature to form light yellow needle-like solid over 24 h. The solid was filtered, rinsed with water (200 mL) and acetone (100 mL), and collected to yield **BAM15** in a quantitative yield. HPLC purity was >98% as determined on a Phenomenex LUNA column (150 mm x 2.0 mm, 5 µm, C18) using a solvent system of 1% formic acid in H_2O (mobile phase A) and 1% formic acid in acetonitrile (mobile phase B) on a Agilent 1100 binary pump with a gradient of 50-95% mobile phase A→B at a flow rate of 0.2 mL/min: retention time = 14.8. ^{1}H NMR (($CD_3)_2CO$, 400 MHz) δ 11.10 (br s, 1H), 9.82 (br s, 1H), 8.68 (br s, 1H), 7.94 (br s, 1H), 7.28 (br, 6H); ^{13}C NMR (101 MHz, ($CD_3)_2CO$) δ 155.1 (d, J = 245.3 Hz),

148.8, 146.8, 130.1 (d, J = 11.6 Hz), 126.7 (d, J = 7.6 Hz), 125.6 (d, J = 3.8 Hz), 124.3 (d, J = 1.5 Hz), 116.2 (d, J = 19.5 Hz); HRMS (ESI+): Calcd for $C_{16}H_{11}F_2N_6O^+$ $[M+H]^+$: 341.0957, Found: 341.0950. Anisotropic displacement ellipsoid drawing (50% probability) of BAM15 is shown below (an acetone solvate is omitted for clarity). CCDC # IS 11979698 contains the supplementary crystallographic data for **BAM15** (CHE-1726077), which can be obtained free of charge via www.ccdc.cam.ac.uk/data_request/cif, or by emailing data_request@ccdc.cam.ac.uk, or by contacting The Cambridge Crystallographic Data Centre, 12 Union Road, Cambridge CB2 1EZ, UK; fax: + 44 1223 336033.

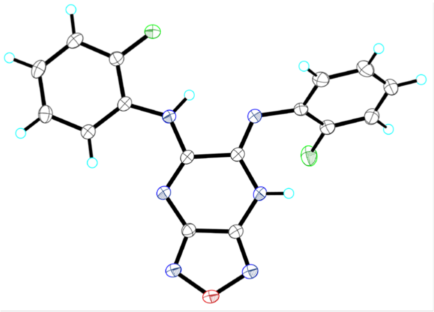

Supplemental Methods: Biology

Western blot analysis

Liver tissue that was sent for metabolomics analysis was also analysed by western blot for phosphorylation of AMPK and its downstream substrate acetyl-CoA carboxylase enzymes. Liver tissue was powdered in liquid nitrogen then homogenized with a motorized pellet pestle homogenizer in PBS containing protease inhibitors (Sigma, S8830-2TAB, Australia) and phosphatase inhibitors (Sigma, P5726-1ML, Australia). Homogenate was probe sonicated for 20 pulses and centrifuged at 20,000 xg for 10 minutes. Supernatant was collected and protein concentration was quantified using the Pierce Bicinchoninic Acid (BCA) Protein Assay Kit (ThermoScientific, 23225, Australia).

Protein lysates were mixed with 5 x Laemmli buffer (250 mM Tris (pH 6.8), 25% RO water 10 % (w/v) SDS, 25 % (v/v) glycerol, 0.2 % w/v bromophenol blue, and 5% (v/v) β-mercaptoethanol) and heated for 5 minutes at 95ºC. Protein lysates (15 μg) were resolved on Any kD™ Mini-Protean TGX Precast gels (Bio-Rad, 4569035, Australia), each sample was resolved on two gels concurrently, and then electro-transferred to nitrocellulose membranes. SeeBlue® Plus2 pre-stained protein standard (Invitrogen, LC5925) was used to determine molecular weight ranges on each gel. Protein transfer was confirmed using Ponceau S staining. Membranes were blocked with 5% (w/v) skim milk in TBST (Tris-buffered saline with 0.1% (v/v) Tween 20) for 1.5 hours.

Nitrocellulose membranes were incubated overnight at 4ºC with antibodies including AMPK (Santa Cruz, sc-74461, USA), phospho-AMPKα (Thr172) (Cell Signaling Technology, 2535, USA), phospho-Acetyl-coA Carboxylase (pACC) (Ser79) (Cell Signaling Technology, 3661, USA), on one membrane, and Acetyl-coA Carboxylase (ACC) (Cell Signaling Technology, 3676, USA) on an second identical membrane. All membranes were incubated with loading control antibody 14-3-3 (Santa Cruz, sc-1657, USA). Membranes were washed three times for 10 minutes in TBST at 4ºC and were then incubated with donkey anti-mouse IgG (AlexaFluor790) (Abcam, ab186699) or anti-rabbit IgG (AlexaFluor680) (Abcam, ab186692) secondary antibodies for 1.5 hours at 4ºC. Membranes were then washed in TBST three times for 10 minutes at 4ºC, then scanned on the LI-COR ODYSSEY CLx System (LI-COR, Lincoln, NE, USA). Densitometry was used to calculate the ratio of phosphorylated protein to total protein.

Supplemental Data

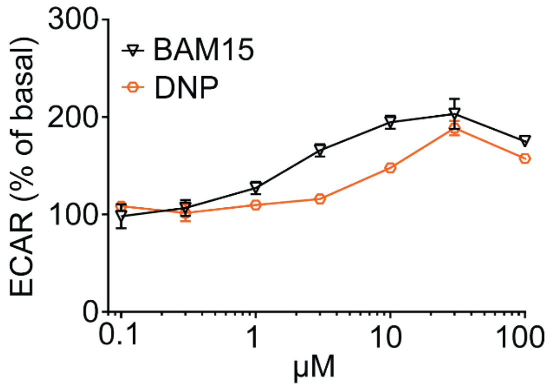

Supplementary Figure 1. Uncoupler-induced change in extracellular acidification rate (ECAR). Normal murine liver cells (NMuLi) were treated with a dose response of DNP or BAM15 at concentrations indicated in the graph. Changes in ECAR were detected in a Seahorse XF96 analyzer and are plotted relative to DMSO control. *n*=3 independent experiments.

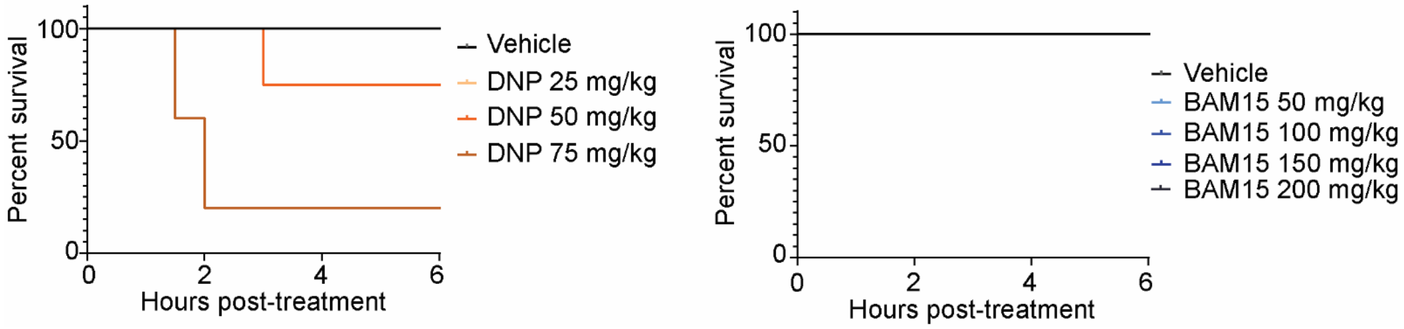

Supplementary Figure 2. Tolerability. Mice were administered an oral gavage of Vehicle control, DNP, or BAM15 at indicated doses. Mice were observed for 6 hours. Mice were culled if signs of ill thrift were observed. DNP and BAM15 experiments were performed separately. For the DNP study *n*=5 animals for Vehicle, 25mg/kg, 75 mg/kg, *n*=4 animals 50 mg/kg. For the BAM15 study *n*=6 animals for all treatments.

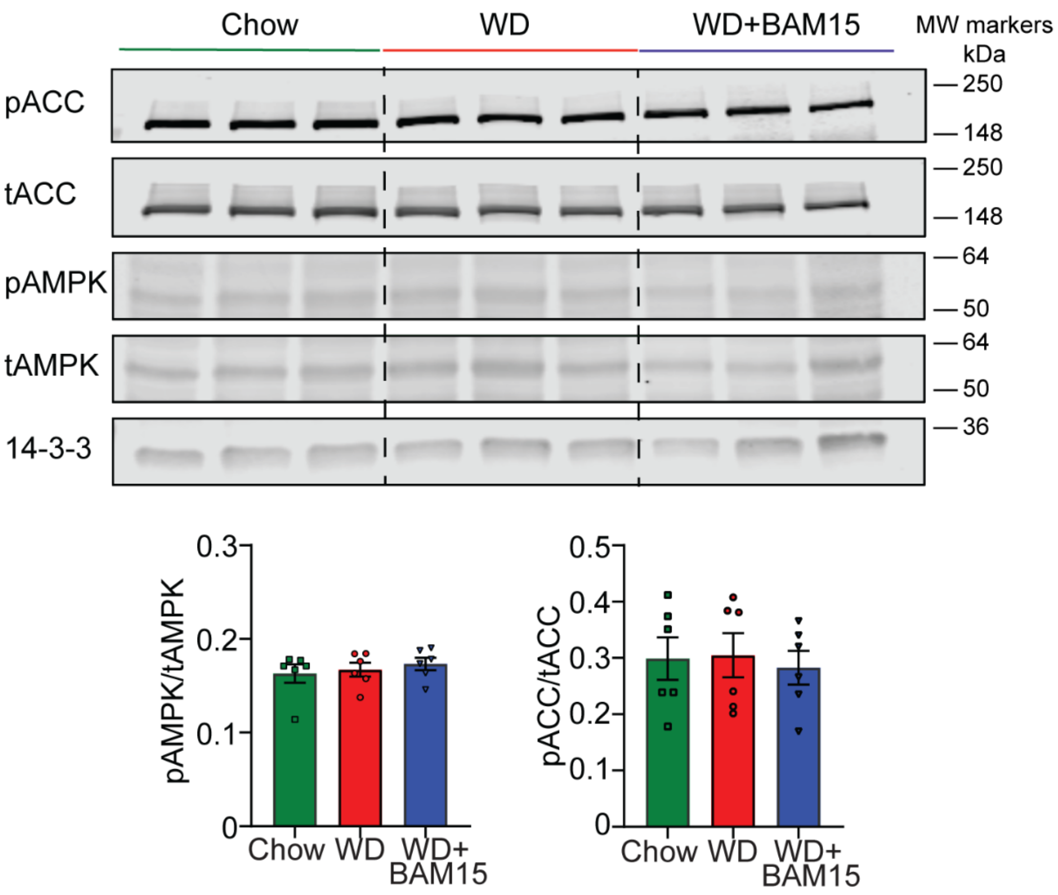

Supplementary Figure 3. Western blot of liver AMPK expression and activity towards ACC enzymes. Liver tissue of mice sacrificed at day 20 (same time point as metabolomics data) of the obesity reversal study design (Figure 4) were analyzed by Western blot for AMPK expression and activity towards its downstream substrate ACC. A representative blot is shown, and densitometry bar graphs represent the averaged data mean ± SEM from all samples, *n*=6 animals for each treatment. Statistical significance was assessed by one-way ANOVA.

Supplementary Table 1 – Serum clinical chemistry and hematology

	Chow	WD	WD+BAM15	*p*-value
**Clinical Chemistry**				
Albumin (g/L)	36.4 ± 0.2	33.2 ± 2.4	35.4 ± 0.8	0.48
ALT (U/L)	40.4 ± 7.0	20.0 ± 3.3	27.0 ± 6.2	0.61
AST (U/L)	95.6 ± 15.2	74.8 ± 18.2	109.6 ± 37.2	0.54
Bicarb (mM)	5.8 ± 0.4	6.2 ± 2.0	4.4 ± 1.3	0.69
β-OH butyrate (mM)	0.2	0.3 ± 0.1	0.2	0.95
BUN (mg/dL)	26.8 ± 1.8	29.1 ± 1.3	35.6 ± 1.1	0.01
Cholesterol (mM)	2.5 ± 0.2	5.3 ± 0.3	4.7 ± 0.3	0.31
Creatine kinase (U/L)	1122 ± 334.2	360.6 ± 42.8	391.6 ± 110.5	0.99
Creatinine (μM)	28.0 ± 4.1	24.0 ± 4.0	26.0 ± 5.6	0.93
GLDH (U/L)	12.8 ± 1.8	12.2 ± 1.4	10.6 ± 1.9	0.74
Globulin (g/L)	10.4 ± 0.9	17.6 ± 2.2	16.8 ± 2.3	0.94
Protein (g/L)	45.0 ± 1.1	50.8 ± 3.7	52.2 ± 2.7	0.91
Triglyceride (mM)	0.9 ± 0.1	2.1 ± 0.2	1.5 ± 0.2	0.04
**Hematology**				
Hb (g/l)	126.0 ± 7.4	137.0 ± 1.6	141.7 ± 4.4	0.83
Hematocrit	0.4	0.4	0.4	0.77
MCH (pg)	14.4 ± 0.2	13.8 ± 0.3	13.7 ± 0.3	0.97
MCHC (g/L)	326.8 ± 1.4	324.8 ± 1.5	320.3 ± 3.7	0.31
MCV (fL)	44.0 ± 0.4	41.8 ± 0.3	43.0 ± 0.6	0.16
Eosinophils ($x10^9$/L)	0.2 ± 0.1	0.3 ± 0.1	0.3 ± 0.1	0.99
Lymphocytes ($x10^9$/L)	6.7 ± 0.5	7.0 ± 0.7	6.9 ± 0.4	0.97
Monocytes ($x10^9$/L)	0.4	0.5	0.4	0.66
Neutrophils ($x10^9$/L)	0.8 ± 0.1	0.8 ± 0.2	0.8 ± 0.1	0.99
Platelets ($x10^9$/L)	1369.8 ± 102.8	1227.8 ± 42.4	1274.7 ± 117.7	0.93
RCC ($x10^{12}$/L)	8.8 ± 0.5	10.2 ± 0.2	10.3 ± 0.4	0.96
WBC ($x10^9$/L)	8.2 ± 0.7	8.5 ± 0.9	8.6 ± 0.3	0.99

Whole blood collected via retro-orbital bleed. Data is presented as mean ± SEM. Significance determined with one-way ANOVA, *p*-value presented refers to BAM15 compared to WD control. *n*=5 animals for all clinical chemistries, hematology performed on *n*=5 Chow, *n*=4 WD and *n*=3 BAM15 animals due to clotting in some samples.

Supplementary Table 2 – Liver N-acetylated amino acid abundance

BIOCHEMICAL	log_2(WD+BAM15/WD)	*p*-value
glycine	0.1	0.538
N-acetylglycine	-0.2	0.512
serine	0.1	0.280
N-acetylserine	-0.9	0.002
threonine	0.1	0.274
N-acetylthreonine	-0.9	0.002
alanine	-0.1	0.364
N-acetylalanine	-0.4	0.090
aspartate	0.2	0.307
N-acetylaspartate (NAA)	0.3	0.230
asparagine	0.0	0.885
N-acetylasparagine	0.1	0.645
glutamate	0.0	0.973
N-acetylglutamate	-0.1	0.856
glutamine	-0.1	0.768
N-acetylglutamine	-0.5	0.129
histidine	-0.1	0.492
1-methylhistidine	-1.1	0.010
N-acetylhistidine	0.2	0.281
lysine	0.0	0.692
N2-acetyllysine	0.2	0.222
phenylalanine	0.1	0.403
N-acetylphenylalanine	-0.2	0.529
tyrosine	0.0	0.879
N-acetyltyrosine	-0.9	0.139
N-formylphenylalanine	-0.4	0.025
leucine	0.1	0.411
N-acetylleucine	-0.6	0.021
isoleucine	0.0	0.682
N-acetylisoleucine	-0.5	0.100
valine	0.1	0.589
N-acetylvaline	-0.8	0.015
methionine	0.1	0.262
N-acetylmethionine	0.5	0.086
cysteine	0.5	0.309
N-acetylcysteine	0.1	0.635
taurine	0.1	0.780
N-acetyltaurine	0.2	0.738
arginine	0.4	0.283
N-acetylarginine	-0.1	0.806
citrulline	0.1	0.477
N-acetylcitrulline	0.4	0.442

Metabolomic analysis was performed on liver tissue collected from animals euthanized on day 20 of treatment. Peak AUC log2 transformed and WD+BAM15/WD fold change calculated. Metabolites color coded by fold -change scale of 2-fold positive (red) to 2–fold negative (blue). *p*-value determined by two tailed Student's t-test. *n*=6 animals from one study.

Supplementary Table 3 – Liver ceramide and diacylglycerol abundance

BIOCHEMICAL	log2(WD+BAM15/WD)	*p*-value
**Ceramides**		
N-palmitoyl-sphingosine (d18:1/16:0)	0.1	0.766
N-stearoyl-sphingosine (d18:1/18:0)	0.0	0.807
N-palmitoyl-sphingadienine (d18:2/16:0)	-0.1	0.306
N-behenoyl-sphingadienine (d18:2/22:0)	-0.5	0.166
N-palmitoyl-heptadecasphingosine (d17:1/16:0)	0.2	0.555
ceramide (d18:1/14:0, d16:1/16:0)	0.2	0.500
ceramide (d18:1/17:0, d17:1/18:0)	0.1	0.689
ceramide (d18:1/20:0, d16:1/22:0, d20:1/18:0)	-0.4	0.074
ceramide (d16:1/24:1, d18:1/22:1)	-0.3	0.356
ceramide (d18:2/24:1, d18:1/24:2)	0.0	0.968
**Diacylglycerols**		
diacylglycerol (14:0/18:1, 16:0/16:1)	-0.7	0.080
diacylglycerol (16:1/18:2 [2], 16:0/18:3	-1.0	0.198
palmitoyl-oleoyl-glycerol (16:0/18:1)	-0.8	0.257
palmitoyl-linoleoyl-glycerol (16:0/18:2)	-1.0	0.262
palmitoyl-linolenoyl-glycerol (16:0/18:3)	-0.5	0.132
palmitoleoyl-oleoyl-glycerol (16:1/18:1)	-1.5	0.058
palmitoleoyl-linoleoyl-glycerol (16:1/18:2)	-0.5	0.320
palmitoyl-arachidonoyl-glycerol (16:0/20:4)	-0.4	0.508
palmitoleoyl-arachidonoyl-glycerol (16:1/20:4)	-0.4	0.364
palmitoyl-docosahexaenoyl-glycerol (16:0/22:6)	-0.6	0.323
oleoyl-oleoyl-glycerol (18:1/18:1)	-0.7	0.312
oleoyl-linoleoyl-glycerol (18:1/18:2)	-0.6	0.369
oleoyl-linolenoyl-glycerol (18:1/18:3)	0.7	0.597
linoleoyl-linoleoyl-glycerol (18:2/18:2)	-0.6	0.442
linoleoyl-linolenoyl-glycerol (18:2/18:3)	-0.1	0.699
stearoyl-arachidonoyl-glycerol (18:0/20:4)	-0.9	0.159
oleoyl-arachidonoyl-glycerol (18:1/20:4)	-0.5	0.242
linoleoyl-arachidonoyl-glycerol (18:2/20:4)	-0.5	0.290
stearoyl-docosahexaenoyl-glycerol (18:0/22:6)	0.0	0.730
linoleoyl-docosahexaenoyl-glycerol (18:2/22:6)	-1.1	0.118

Lipidomic analysis was performed on liver tissue collected from animals euthanized on day 20 of treatment. Peak AUC log2 transformed and WD+BAM15/WD fold change calculated. *p*-value determined by two-tailed Student's t-test. *n*=6 animals from one study.

Supplementary Table 4 – Fasting and insulin clamp characteristics

	Chow	WD	WD+BAM15
n	9	7	8
**Blood Glucose (mM)**			
**Basal**	7.49 ± 0.27*	8.68 ± 0.38	8.02 ± 0.49
**Clamp**	7.78 ± 0.21	7.71 ± 0.16	7.78 ± 0.22
**Insulin (μU/mL)**			
**Basal**	20.65 ± 2.45*	42.42 ± 7.91	30.83 ± 5.02
**Clamp**	114.83 ± 6.45	122.80 ± 6.72	142.40 ± 9.30
**NEFA (mM)**			
**Basal**	0.84 ± 0.1	0.95 ± 0.05	0.97 ± 0.07
**Clamp**	0.27 ± 0.04*	0.52 ± 0.04	0.37 ± 0.04

Results expressed as mean ± SEM. * indicates p<0.05 compared to WD by two-way RM ANOVA. Exact p-values as follows – Basal blood glucose: Chow, p=0.0170; Basal insulin: Chow, p=0.0429; Clamp NEFA: Chow, p=0.0181.

Supplementary References

1. Kenwood, B.M., Calderone, J.A., Taddeo, E.P., Hoehn, K.L. & Santos, W.L. Structure-activity relationships of furazano[3,4-b]pyrazines as mitochondrial uncouplers. *Bioorg. Med. Chem. Lett.* **25**, 4858-4861 (2015).
2. Fernández, E.*, et al.* Solid-phase versus solution synthesis of asymmetrically disubstituted furazano[3,4-b]pyrazines. *Tetrahedron Letters* **43**, 4741-4745 (2002).
3. Starchenkov, I.B. & Andrianov, V.G. Chemistry of furazano[3,4-b]pyrazines. *Chemistry of Heterocyclic Compounds* **33**, 1219-1233 (1997).
4. Thottempudi, V., Yin, P., Zhang, J., Parrish, D.A. & Shreeve, J.n.M. 1,2,3-Triazolo[4,5,-e]furazano[3,4,-b]pyrazine 6-Oxide—A Fused Heterocycle with a Roving Hydrogen Forms a New Class of Insensitive Energetic Materials. *Chemistry – A European Journal* **20**, 542-548 (2014).
