# Supplementary material for: Mitochondrial uncoupler BAM15 reverses diet-induced obesity and insulin resistance in mice
Source: Nat Commun. 2020 May 14;11:2397. doi: 10.1038/s41467-020-16298-2 (PMC7224297; doi:10.1038/s41467-020-16298-2)
Supplement: Supplementary file 2 — Description of Additional Supplementary Files [file 41467_2020_16298_MOESM2_ESM.docx]

Description of Additional Supplementary Files

**Supplementary Dataset 1:** Metabolomics analysis performed by Metabolon Inc. conducted on liver tissue following 20 days of treatment with 0.1% w/w BAM15 in Western Diet (WD+BAM15) or WD diet control (WD). Data is reported as peak area under the curve (AUC) for each metabolite, for each animal. n=6 animals from one study.
