# Supplementary material for: Mitochondrial uncoupler BAM15 reverses diet-induced obesity and insulin resistance in mice
Source: Nat Commun. 2020 May 14;11:2397. doi: 10.1038/s41467-020-16298-2 (PMC7224297; doi:10.1038/s41467-020-16298-2)
Supplement: Supplementary file 4 — Reporting Summary [file 41467_2020_16298_MOESM4_ESM.pdf]

nature research

Corresponding author(s): Kyle Hoehn

Last updated by author(s): 3 April 2020

Reporting Summary

Nature Research wishes to improve the reproducibility of the work that we publish. This form provides structure for consistency and transparency in reporting. For further information on Nature Research policies, see Authors & Referees and the Editorial Policy Checklist .

Statistics

For all statistical analyses, confirm that the following items are present in the figure legend, table legend, main text, or Methods section.

n/a	Confirmed	
☐	☒	The exact sample size (*n*) for each experimental group/condition, given as a discrete number and unit of measurement
☐	☒	A statement on whether measurements were taken from distinct samples or whether the same sample was measured repeatedly
☐	☒	The statistical test(s) used AND whether they are one- or two-sided<br>*Only common tests should be described solely by name; describe more complex techniques in the Methods section.*
☒	☐	A description of all covariates tested
☐	☒	A description of any assumptions or corrections, such as tests of normality and adjustment for multiple comparisons
☐	☒	A full description of the statistical parameters including central tendency (e.g. means) or other basic estimates (e.g. regression coefficient) AND variation (e.g. standard deviation) or associated estimates of uncertainty (e.g. confidence intervals)
☐	☒	For null hypothesis testing, the test statistic (e.g. *F*, *t*, *r*) with confidence intervals, effect sizes, degrees of freedom and *P* value noted<br>*Give P values as exact values whenever suitable.*
☒	☐	For Bayesian analysis, information on the choice of priors and Markov chain Monte Carlo settings
☒	☐	For hierarchical and complex designs, identification of the appropriate level for tests and full reporting of outcomes
☒	☐	Estimates of effect sizes (e.g. Cohen's *d*, Pearson's *r*), indicating how they were calculated

Our web collection on statistics for biologists contains articles on many of the points above.

Software and code

Policy information about availability of computer code

Data collection	Metabolon's Laboratory Information Management System (LIMS)
Data analysis	Metabolon's Laboratory Information Management System (LIMS), R (v.3.5.5) and Prism (v.8.1.2) GraphPad Software, Shimazdu LabSolutions Software.

For manuscripts utilizing custom algorithms or software that are central to the research but not yet described in published literature, software must be made available to editors/reviewers. We strongly encourage code deposition in a community repository (e.g. GitHub). See the Nature Research guidelines for submitting code & software for further information.

Data

Policy information about availability of data

All manuscripts must include a data availability statement. This statement should provide the following information, where applicable:

- Accession codes, unique identifiers, or web links for publicly available datasets
- A list of figures that have associated raw data
- A description of any restrictions on data availability

All data included in this publication has been provided in the Source data file. Additional data that support the findings of this study are available from the corresponding author upon reasonable request.

Field-specific reporting

Please select the one below that is the best fit for your research. If you are not sure, read the appropriate sections before making your selection.

Life sciences study design

All studies must disclose on these points even when the disclosure is negative.

Sample size	Sample size estimates were gated on the number of animals needed to see a 50% decrease in weight gain with a standard deviation of 10-15% of the mean, in both obesity prevention and reversal studies. Using calculations with alpha 0.05 and power 80%, the required sample size was calculated to be 6 mice per group. Sample sizes of >6 were used for most experiments.
Data exclusions	In two cases data from the hyperinsulinemia-euglycemic clamp study was excluded for technical reasons including animals that did not recover from dual catheter surgery prior to hyperinsulinemia-euglycemic clamp, as well as erratic/outlier values obtained due to a loose catheter or hyperactivity of the animal. In addition, during an i.p. GTT data was excluded due to a mis-injection of glucose solution into intestine that was evidenced by no elevation in glucose 15 minutes post-injection. No data or animals were excluded due in other experiments, and no experiments were terminated prematurely.
Replication	Reversal and prevention studies at 0.01% BAM15 in Western Diet have been replicated by our laboratory. The replicated studies have been combined and included in this publication. The prevention study has been repeated three times with similar results. The reversal study has been repeated six times, for different lengths of study duration, with similar results. Seahorse experiments have been repeated by our laboratory in excess of twenty times with similar findings. All other studies such as the Oxymax CLAMS experiments have not been repeated.
Randomization	Mice were stratified to ensure body composition and baseline glucose tolerance were matched across treatment groups then randomly assigned to treatment groups.
Blinding	Blinding was not possible for most experiments as group allocation and treatment was administered by the researcher collecting the data. However, for histological examination samples were de-identified and therefore blinded to the pathologist performing the examination.

Reporting for specific materials, systems and methods

We require information from authors about some types of materials, experimental systems and methods used in many studies. Here, indicate whether each material, system or method listed is relevant to your study. If you are not sure if a list item applies to your research, read the appropriate section before selecting a response.

Materials & experimental systems

n/a	Involved in the study	
☐	☒	Antibodies
☐	☒	Eukaryotic cell lines
☒	☐	Palaeontology
☐	☒	Animals and other organisms
☒	☐	Human research participants
☒	☐	Clinical data

Methods

n/a	Involved in the study	
☒	☐	ChIP-seq
☒	☐	Flow cytometry
☒	☐	MRI-based neuroimaging

Antibodies

Antibodies used	AMPK (Santa Cruz, sc-74461, USA), phospho-AMPKα (Thr172) (Cell Signaling Technology, 2535, USA), Acetyl-coA Carboxylase (ACC) (Cell Signaling Technology, 3676, USA), Acetyl-coA Carboxylase (ACC) (Cell Signaling Technology, 3676, USA), phospho-ACC (Ser79) (Cell Signaling Technology, 3661, USA) and 14-3-3 (Santa Cruz, sc-1657, USA)
Validation	The following antibodies have previously been used by our laboratory for the detection if the relevant proteins in mouse liver tissue. As per the manufacturers website - AMPK (Santa Cruz, sc-74461, USA) is recommended by the manufacturer for detection of AMPKα1 and AMPKα2 of mouse protein by western blotting.<br>As per the manufacturers website - Phospho-AMPKα (Thr172) (40H9) Rabbit mAb detects endogenous AMPKα only when phosphorylated at threonine 172. The antibody detects both α1 and α2 isoforms of the catalytic subunit. It is appropriate for western blot analysis of mouse tissue.<br>As per the manufacturers website - Acetyl-CoA Carboxylase (C83B10) Rabbit mAb detects endogenous levels of all isoforms of acetyl-CoA carboxylase protein. It is appropriate for western blot analysis of mouse tissue.<br>As per the manufacturers website - Phospho-Acetyl-CoA Carboxylase (Ser79) Antibody detects endogenous levels of ACC only when phosphorylated at serine 79. The antibody recognizes both ACCalpha and ACCbeta. It is appropriate for western blot analysis of mouse tissue.<br>As per the manufacturers website -14-3-3 (Santa Cruz, sc-1657, USA). pan 14-3-3 Antibody (H-8) is a mouse monoclonal IgG2b (kappa light chain)is specific for an epitope mapping between amino acids 1-30 at the N-terminus of 14-3-3 β of human origin. pan 14-3-3 Antibody (H-8) is recommended for detection of pan 14-3-3 of mouse protein by western blot.

Eukaryotic cell lines

Policy information about cell lines

Cell line source(s)	NMuLi cell
Authentication	This cell line has not been authenticated.
Mycoplasma contamination	This cell line has been tested for mycoplasma contamination and found to be negative.
Commonly misidentified lines (See ICLAC register)	*Name any commonly misidentified cell lines used in the study and provide a rationale for their use.*

Animals and other organisms

Policy information about studies involving animals; ARRIVE guidelines recommended for reporting animal research

Laboratory animals	Male C57BL/6J mice. Mice were 2 months of age for each study, with the exception of the obesity prevention study where mice were 4 months of age.
Wild animals	The study did not involve wild animals.
Field-collected samples	The study did not involves samples collected from the field.
Ethics oversight	Mouse experiments were approved by the UNSW Animal Care and Ethics Committee (project approval numbers 14-33A, 17-66B and 18/91A) or where relevant the Garvan Institute of Medical Research (project approval number 18/19).

Note that full information on the approval of the study protocol must also be provided in the manuscript.
